# Characterizing functional modules in the human thalamus: coactivation-based parcellation and systems-level functional decoding

**DOI:** 10.1007/s00429-022-02603-w

**Published:** 2022-12-22

**Authors:** Ole J. Boeken, Edna C. Cieslik, Robert Langner, Sebastian Markett

**Affiliations:** 1 Faculty of Life Sciences, Department of Molecular Psychology, Humboldt-Universität Zu Berlin, Rudower Chaussee 18, 12489 Berlin, Germany; 2 Institute of Systems Neuroscience, Medical Faculty, Heinrich Heine University Düsseldorf, Düsseldorf, Germany; 3 Institute of Neuroscience and Medicine (INM-7: Brain and Behaviour), Research Centre Jülich, Jülich, Germany

**Keywords:** Thalamus, Systems-level decoding, Structure–Function relationships, BrainMap database, ALE meta-analysis, Neurosynth

## Abstract

The human thalamus relays sensory signals to the cortex and facilitates brain-wide communication. The thalamus is also more directly involved in sensorimotor and various cognitive functions but a full characterization of its functional repertoire, particularly in regard to its internal anatomical structure, is still outstanding. As a putative hub in the human connectome, the thalamus might reveal its functional profile only in conjunction with interconnected brain areas. We therefore developed a novel systems-level Bayesian reverse inference decoding that complements the traditional neuroinformatics approach towards a network account of thalamic function. The systems-level decoding considers the functional repertoire (i.e., the terms associated with a brain region) of all regions showing co-activations with a predefined seed region in a brain-wide fashion. Here, we used task-constrained meta-analytic connectivity-based parcellation (MACM-CBP) to identify thalamic subregions as seed regions and applied the systems-level decoding to these subregions in conjunction with functionally connected cortical regions. Our results confirm thalamic structure–function relationships known from animal and clinical studies and revealed further associations with language, memory, and locomotion that have not been detailed in the cognitive neuroscience literature before. The systems-level decoding further uncovered large systems engaged in autobiographical memory and nociception. We propose this novel decoding approach as a useful tool to detect previously unknown structure–function relationships at the brain network level, and to build viable starting points for future studies.

**Supplementary Information:**

The online version contains supplementary material available at 10.1007/s00429-022-02603-w.

## Introduction

The human thalamus is known to participate in sensorimotor (Herrero et al. 2002; Saalmann and Kastner 2011) and as well in a variety of higher cognitive functions such as reward (Haber and Knutson 2010), language (Llano 2013), attention (Schmitt et al. 2017), executive (Marzinzik et al. 2008; Varela 2014; Halassa and Kastner 2017), and memory processes (Pergola et al. 2013; Carlesimo et al. 2015). The thalamus is, however, not a unitary structure, and its segregated nature determines its behavioral and cognitive characteristics (Sherman et al. 2006; Sherman 2016). Traditionally, the thalamic nuclei were delineated based on histological features (Vogt and Vogt 1941; Morel et al. 1997; Krauth et al. 2010). Modern (network) neuroimaging has refined these segmentations by investigating the wide-spread connections of the thalamus and implicated the thalamus in a variety of cognitive functions through its reciprocal interconnections with cerebellar, subcortical, and cortical neural circuits (Behrens et al. 2003; Johansen-Berg et al. 2005; Moustafa et al. 2017). However, from a (cognitive) neuroscience perspective, the exact internal functional organization of the thalamus and its subdivisions has not been fully elucidated. There are several reasons for this as follows: Thalamic nuclei participate in a highly diverse set of cortical systems, while the connectivity patterns of some nuclei seem to overlap, which comes with a heterogeneous picture regarding the functional properties of the individual nuclei (Yuan et al. 2016), further complicating the functional characterization of thalamic nuclei. In this sense, thalamic nuclei that relay sensorimotor information show rather specific functions (Sherman 2016). In contrast, nuclei that are supposed to actively process information from different cortical systems, are more flexible in their participation in a broad range of cognitive functions (Fama and Sullivan 2015; Mitchell 2015; Yuan et al. 2017; Wolff and Vann 2019; Antonucci et al. 2021). The required systematic application of a variety of experimental paradigms to further disentangle the functions of brain regions is, however, constrained by practical factors such as the high costs and the tremendous effort involved in organizing (large-scale) functional neuroimaging studies (Eickhoff et al. 2011).

The functional characterization of brain regions is facilitated by meta-analytic decoding using databases such as BrainMap (Laird et al. 2011) or Neurosynth (Yarkoni et al. 2011) that comprise the data of thousands of neuroimaging studies, tapping into a variety of psychological constructs. An assessment of the functional repertoire of a particular pre-defined brain area requires a well-elaborated choice of an atlased parcellation. In the literature, many different brain parcellation schemes have been proposed to delineate regional boundaries based on different structural and functional properties. Oftentimes, distinct nomenclatures are used in these efforts to designate the regions observed, challenging the integration of neuroimaging results (Bohland et al. 2009; Amunts et al. 2014; Eickhoff et al. 2018; Salehi et al. 2020). This is especially true for the thalamus, for which several parcellations exist, each with a different level of detail (Mai and Majtanik 2019; Iglehart et al. 2020). Previous thalamic parcellations delineated, for instance, either seven (Behrens et al. 2003; Najdenovska et al. 2018), 9 (Hwang et al. 2017), 11 (Saranathan et al. 2021), 15 (Kumar et al. 2017), 21 (Kumar et al. 2015), 25 (Iglesias et al. 2018), or 38 distinct nuclei in the Morel atlas (Krauth et al. 2010; Morel et al. 1997). For the particular purpose of meta-analytic decoding, however, the existing thalamic parcellations may be not well-suited. Parcels need to match the spatial resolution of the cognitive neuroscience experiments in the data bases. Quite likely, histology-based parcellations (Krauth et al. 2010; Morel et al. 1997) are too fine-grained, since they feature a large number of small nuclei which do not match the still relatively low spatial resolution of functional neuroimaging (Traynor et al. 2010; Steullet 2020). Furthermore, parcels need to match the functional resolution of task-constrained co-activations. Even though task activity is ultimately supported by structural and functional connectivity (Tavor et al. 2016; Cole et al. 2016), parcels derived from either anatomical (Behrens et al. 2003; Najdenovska et al. 2018; Saranathan et al. 2021) or (resting-state) functional connectivity (Kumar et al. 2017; Hwang et al. 2017) are not guaranteed to carry task-related information (Eickhoff et al. 2011).

We therefore aimed to build a novel data-driven parcellation of the human thalamus, based on brain-wide meta-analytic connectivity modeling (MACM; Eickhoff et al. 2011; Langner and Camilleri 2021)) using the co-activation patterns of thousands of functional neuroimaging experiments stored in the BrainMap database (Laird et al. 2011). The associated connectivity-based parcellation (MACM-CBP) technique has been previously applied to parcellate the dorsolateral prefrontal cortex (Cieslik et al. 2013), the pulvinar (Barron et al. 2015), the frontal pole (Ray et al. 2015), the left and right premotor cortices (Genon et al. 2016, 2018b), or the ventromedial frontal lobe (Chase et al. 2020). The usage of such data-driven parcellations facilitates the testing of hypotheses on the behavior (i.e., the cognitive-functional profile) of the newly derived subunits with Bayesian reverse inference decoding. By adapting this decoding strategy, we were able to aggregate data of several thousand neuroimaging studies and probe the functional implications of different thalamic regions in different behavioral contexts.

In the present research we aimed to characterize the function of thalamic nuclei, derived via MACM-CBP, with reverse inference decoding based on data stored in the Neurosynth database (Yarkoni et al. 2011). Instead of using the BrainMap database as source for the functional characterization, as done in previous decoding studies, we here used Neurosynth because it includes a larger corpus of neuroimaging datasets. Furthermore, Neurosynth contains a set of functional terms that are a product of automated data-driven text-mining, which provides a larger variety of possible terms for functional associations. Considering the dense interconnectedness of the thalamus with cortical regions, we extended the established decoding strategy with a new approach, which we propose is more sensitive to uncover thalamic functional organization, as compared to already existing decoding strategies. The novel approach—which we call systems-level decoding—decodes structure–function associations simultaneously for pairs of brain regions (here: individual MACM-CBP-derived thalamic seed regions and functionally coupled cortical regions) and retains those functions that are not already associated with either of the two regions alone. A precursor systems-level decoding approach has previously been used successfully to characterize clusters of brain regions involved in cognitive action or emotion regulation (Langner et al. 2018) and here we aimed to expand the knowledge about the functional spectrum of thalamic nuclei, acknowledging the fact that brain regions work in concert to process stimuli and mediate behaviors.

## Methods

### Definition of thalamic seed regions

We used the Oxford thalamic connectivity atlas template (Behrens et al. 2003) to define the seed volume. The Oxford atlas is based on structural data, which circumvents the problem of circularity when examining structure–function associations (Kriegeskorte et al. 2009). The thalamic mask was split along the midline for the left and the right hemisphere, thresholded and binarized (≥ 25% probability) using FSL (Jenkinson et al. 2012). The left mask comprised 1321 voxels with 2 × 2 × 2 mm^3^ resolution, the right mask comprised 1283 voxels.

### Meta-analytic connectivity modeling (MACM) and connectivity-based parcellation (CBP)

The two thalamic seed regions were parcellated based on the task-derived whole-brain co-activation patterns of all voxels in the respective thalamic mask. To determine the co-activation pattern for each voxel within the left and right thalamic masks, we queried the BrainMap database (Laird et al. 2011) and performed coordinate-based meta-analysis on the output. Inclusion criteria of the database query required eligible experiments to be *normal mapping* studies using fMRI or PET with *healthy adults* and reporting task-related *activations*. Thus, neither intervention studies nor clinical or developmental studies were included in the analysis. From all 7937 eligible experiments in the database, we then associated experiments with each voxel independently to construct co-activation maps for each voxel. When it comes to the association between seed voxels and experiments within the BrainMap database, the number of studies reporting activation precisely at a seed voxel might be low or variable (Bzdok et al. 2013; Ray et al. 2015). We therefore selected the experiments associated with each voxel, from studies that showed activation at or in the immediate neighborhood to that voxel. That is, in steps of two, we applied a (spatial) filter to get the 20–200 (i.e., yielding 91 filter sizes in total) experiments that reported the closest activation to the current seed voxel. As done in previous studies, the closeness was defined by calculating the Euclidean distances between the current seed voxel and the foci of all experiments that met the specified (search) criteria. This method has proven to provide a reliable basis for MACM-CBP, as demonstrated in former studies (Cieslik et al. 2013; Clos et al. 2013).

Then, for each seed voxel, the brain-wide co-activation pattern for each of the 91 filter sizes was computed by activation likelihood estimation (ALE) meta-analysis (Eickhoff et al. 2012) across all experiments associated with the given voxel (i.e., reporting activation at or near this voxel, as described above). The core principle of the ALE approach is to treat reported activation foci not as absolute points in space but as 3D Gaussian probability distributions, to account for spatial uncertainties of functional imaging data (Turkeltaub et al. 2002; Eickhoff et al. 2009). Probability distributions of all reported foci were combined in a modeled activation map for each experiment associated with a particular seed voxel. Then, the voxel-wise union across the activation maps of all experiments associated with the given seed voxel yielded an ALE score for each voxel of the brain. The ALE score describes the probability of a co-activation with the given seed voxel. At this point of the analysis, no thresholding was applied, to preserve the full quantitative repertoire of whole-brain co-activation patterns for the later parcellation of the thalamic masks in an unconstrained manner. This procedure yielded a N_s_ × N_B_ connectivity matrix, where N_S_ represents the number of seed voxels (i.e., left thalamic mask = 1321, right thalamic mask = 1283) and N_B_ the number of target voxels in the reference brain (26,549 voxels located within the gray matter). As a connectivity matrix was computed for every individual filter size, this procedure resulted in 91 connectivity matrices.

### k-means clustering

To group co-activation patterns into homogenous clusters (“parcels”), we applied k-means clustering to the connectivity matrices individually, as done in previous parcellation studies (Genon et al. 2016; Plachti et al. 2019; Chase et al. 2020). The thalamic masks (i.e., the seed regions) were iteratively divided into a pre-specified number of *k* = 2:8 non-overlapping clusters. The goal of the clustering is to group voxels together that show the greatest possible similarity regarding their co-activation patterns. Here, similarity is defined by minimizing the variance within clusters and maximizing the variance between clusters. Depending on the pre-specified number of k clusters, k centroids are randomly placed, and voxels are assigned to a given centroid.

We explored 7 different clustering solutions with k ranging from 2 to 8 possible clusters. This range was motivated by two points: from a functional perspective, the thalamus might comprise two subdivisions spanning ventral relay areas (e.g., lateral geniculate nuclei (LGN), medial geniculate nucleus (MGN), ventral lateral nucleus (VA)) and midline/dorsal association areas (e.g., mediodorsal nucleus (MD), anterior nucleus (A) (Vertes et al. 2015). Furthermore, we expected that with more than 8 clusters, the derived subunits would be too small to find meaningful functional associations, given the spatial resolution of typical neuroimaging studies.

### Selection of optimal filter range

As done in previous studies (Clos et al. 2013; Genon et al. 2016), the selection of the optimal filter range was based on consistency of each voxel´s cluster assignment across the different filter sizes. That is, the filter range comprising the lowest number of deviants was identified and all following analyses were restricted to these filter sizes. For the left thalamus, optimal filter size ranged from 104 to 156 experiments, and for the right thalamus from 106 to 154 experiments (see supplementary Figure S1).

### Data-driven selection of the optimal clustering solution

Selection of the optimal range for the k-means clustering was based on topological, information-theoretic and cluster separation characteristics. Topological criteria included the *detection of the percentage of misclassified voxels* (i.e., deviants), *voxels not related to the dominant parent cluster* (i.e., related to the hierarchy index), and the *number of consistent voxels per cluster*. The information-theoretic criterion was the *variation of information between the filter sizes.* Cluster separation criteria included the *information on cluster separation* (i.e., the intercluster-to-intracluster distance ratio)*,* the *silhouette value* (i.e., a measure of how similar a given voxel is to voxels in its own cluster compared to voxels in other clusters). A full description and a figure for illustration is given in the supplementary information (S2, S3).

We used the canonical Morel atlas as a well-established thalamic atlas scheme for anatomic labeling. That is, we calculated the degree of overlap of the MACM-CBP-derived parcels with the thalamic nuclei as defined in the Morel atlas by means of the Jaccard Index (Steen et al. 2011), to gain meaningful labels for our parcellation in accordance with a classical view on thalamic demarcation.

### resting-state fMRI data acquisition

As we aimed to characterize thalamic functional properties in a system-wide fashion, we identified cortical regions functionally connected to the MACM-CBP derived thalamic clusters by investigating ﻿﻿﻿﻿﻿the clusters’ individual RSFC pattern. We based the RSFC analysis of thalamic subregions on data from *n* = 84 healthy participants (mean age = 26.23, SD = 5.41 years, 40 females, 44 males). We adopted MRI sequences from the HCP-Lifespan project (Harms et al. 2018) and acquired a T1-weighted structural image (Multiecho MPRAGE, voxel size 0.8 mm isotropic, TR = 2.4 s, TE = 22 ms, flip angle 8°), a T2-weighted structural image (SPACE, voxel size 0.8 mm isotropic, TR = 3.2 s, TE = 563 ms, flip angle 120°), BOLD fMRI (multiband echo-planar imaging, 72 slices, 805 volumes, TR = 800 ms, voxel size 2 mm isotropic, TE = 37 ms, flip angle 52°, A-P encoding direction), as well as two spin-echo fieldmaps (A-P and P-A encoding).﻿﻿﻿﻿﻿

### Resting-state fMRI data preprocessing

We utilized the HCP minimal preprocessing pipelines (github.com/Washington-University/HCPpipelines) for structural and functional preprocessing (Glasser et al. 2013). A detailed description of resting-state data preprocessing is given in Markett et al. (2022). In brief, resting-state fMRI data were corrected for gradient distortions, motion, EPI image distortions, co-registered with the T1 structural image, and normalized to MNI volumetric space based on cortical folding (MSMsulc, (Robinson et al. 2018). Intensity-normalized functional images were further processed with the fMRISurface pipeline to create individual CIFTI dense time-series gray-ordinate files. We applied light volume- and surface-based smoothing with a Gaussian filter with 2 mm full width at half maximum. For artifact removal, resting-state time-series data were first run through FSL’s Multivariate Exploratory Linear Optimized Decomposition into Independent Components (MELODIC) tool (ve3.15) and then processed using FSL Fix (v1.06.15). We used a classifier that had been trained on the HCP young adult sample as distributed with FIX. Artifactual components were regressed out together with the six head motion parameters and their first derivatives. The cleaned fMRI time series data were then converted to gray-ordinate files as described above.

### RSFC analysis

Multivariate functional connectivity analyses were performed for each thalamic subregion by extracting the mean time course from each subregion, residualizing each time course from the other subregion’s time courses, and computing seed-region RSFC maps by running the first stage of FSL’s dual regression. We used the Sandwich Estimator (SwE) Toolbox for SPM12 (Guillaume et al. 2014) to create group-level RSFC maps, applying the modified SwE procedure with a small sample size correction (type c) and a wild bootstrapping procedure with 999 bootstraps. The family-wise error was corrected at the voxel level (*p* < 0.05).

We overlaid each thresholded seed-connectivity map with Glasser et al.’s (2016) multimodal parcellation (HCP-MMP) to identify cortical regions of interest with at least 80% of coverage by the seed-connectivity map (for a complete list of all HCP-MMP-regions, see supplementary Table S4 and supplementary Figure S5). This resulted in thalamic-cortical pairs that later we used for the systems-level decoding. HCP-MMP is a group parcellation that is based on structural and functional (task and rest) imaging data and has been shown to generalize well across samples (Glasser et al. 2016).

### Functional decoding of thalamic clusters

For the functional characterization of the MACM-CBP-derived thalamic parcels, we used the NeurosynthDecoder implemented in the Neuroimaging Meta-Analysis Research Environment (NiMARE; (Salo, Taylor et al. 2021)), which is a Python package for conducting neuroimaging meta-analyses. The Neurosynth database provides a dataset of roughly 3000 terms, covering psychological constructs (e.g., ‘working memory’, ‘episodic memory’), anatomical (e.g., ‘thalamus’, ‘thalamic’) or technical terms (e.g., ‘BOLD’, ‘fMRI’) as obtained from automated parsing through published neuroimaging studies (Yarkoni et al. 2011). An important feature of the NeurosynthDecoder is that it allows for reverse inference decoding of activation-term associations. We can infer the probability by which a given term was over-represented in studies reporting activation at a given location, which gives us an idea which behavior may have been executed while a given brain region was active.

Reverse inference decoding can be applied to arbitrary brain regions and results in estimates of the posterior probability of a term given activation of the region and given the prior probability of a term (i.e., the prevalence of the term in the database). Formally, the posterior probability is computed as $$P(term|activation, p) = p(P(activation|term)/P(activation|term, p)$$*.* Here the prior probability *p* is set beforehand to 0.5 (i.e., 50% chance of a brain region experiencing the brain state described by the term). This rather conservative approach is used to equate possible baserate differences of terms within the database, which in turn should facilitate the interpretation of the large number of (otherwise very different) posterior probabilities (Yarkoni et al. 2011). For clarification, $$P(activation|term,p)$$ represents the forward inference and gets computed as $$P(activation|term,p) = pP(activation|term) + (1-p)P(activation|not having the term).$$ In addition to reporting the posterior probabilities that can be interpreted as effect sizes of the reverse inference decoding, a two-way chi-square test was performed to determine if the presence of the label and the selection of a term are statistically independent (alpha < 0.05). Multiple comparison correction is done by applying a Benjamini–Hochberg FDR correction. Multiple comparison correction was applied for each cluster separately. The number of multiple comparisons depends on the number of terms stored in the database (For details, see the NIMARE documentation, accessible at: https://www.nimare.readthedocs.io/en/latest/decoding.html).

In this study, we submitted each MACM-CBP-derived cluster individually to the NeurosynthDecoder. This resulted in a list of associated terms and their posterior probabilities. Of these terms, we selected the ten terms with the highest posterior probability for each cluster for the sake of comparability. The automatic NeurosynthDecoder lists all terms, irrespective of whether or not they passed the multiple-comparison significance testing. We therefore excluded all terms that did not pass the multiple-comparison correction post hoc using an in-house MATLAB (MATLAB 2021) script.

### Systems-level decoding of thalamic clusters

Brain regions work together to process stimuli and realize behavior. Especially regions that serve as important hub regions in brain networks are related to a diverse set of functions through their large number of connections (van den Heuvel and Sporns 2013; Margulies et al. 2016; Bertolero et al. 2017).

Evidence shows that the thalamus, as a densely interconnected brain region, serves as such an important hub region that integrates multimodal signals from functional brain systems (Crossley et al. 2013; Hwang et al. 2017). Consequently, we applied a novel systems-level decoding approach to the MACM-CBP-derived thalamic clusters and their respective functionally connected regions.

**Fig. 1:**
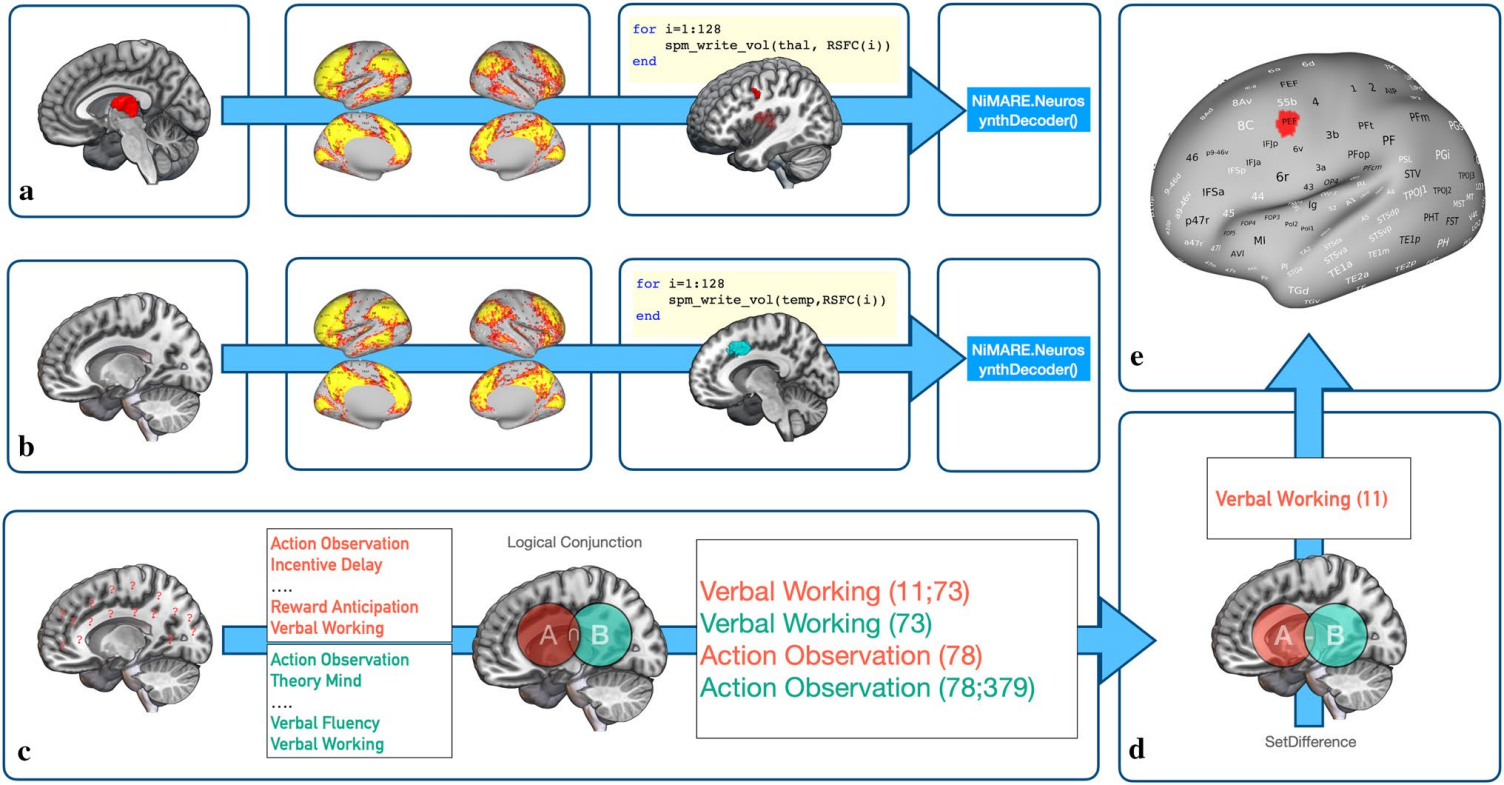
Steps of the systems-level decoding illustrated with an example for the MACM-CBP-derived left cluster 1. **a** In the first step of the systems-level decoding, we generated individual thalamic-cortical masks for the insertion into the NeurosynthDecoder. This process was repeated for all cortical regions that were functionally connected at rest with the respective thalamic cluster. In the example, the left cluster 1 is functionally connected to 128 regions. For reasons of legibility, however, only the mask of the left cluster 1 and the region 11 of the HCP-MMP (i.e., the left Premotor Eye Field) are displayed here. **b** We repeated the decoding for all masks of the functionally connected regions only. In the example above the mask of region 73 (i.e., left Area C in the Dorsolateral Prefrontal cortex) is displayed. **c** In the next step, we calculated the intersect between the terms from the two decoding runs with a logical AND conjunction. Note, for the reason of legibility, we here only display a limited selection of terms. **d** At the final stage of the analysis, regional IDs were used as the basis for the calculation of the set difference between the two sets. In the example above, the only term that surpasses the two filtering steps is the term ‘verbal working’ associated with the left Premotor Eye Field. **e** Based on all leftover regional-term associations, we created cifti files, to display the ‘systems” that were associated with the generation of a given term across the brain. Cortical renderings were created with MRIcroGL (Rorden and Brett 2000) and surface maps with connectome workbench commands (Marcus et al. 2011)

The basic rules for the generation of reverse inference maps for the systems-level decoding were the same as for the decoding of individual thalamic clusters. At this step of the analysis, however, we decoded all thalamic clusters together with their respective functionally connected regions in thalamo-cortical pairs (see Fig. 1 for details). More specifically, the mask of a given thalamic cluster was merged with the foci from an HCP-MMP-region that was functionally connected with that thalamic cluster (i.e., *p* < 0.05, FWE-corrected, with at least 80% coverage) to create individual volumetric images (i.e., masks) for all thalamo-cortical pairs. After decoding, we combined the top-10 terms of all thalamo-cortical pairs into a large dataset, filtered out regions that did not pass the multiple-comparison correction (pReverse < 0.05), and tagged each remaining term with the respective HCP-MMP regional ID (see Fig. 1A). The numeric tags reflect the regional ID from the HCP-MMP parcellation to make sure that we were later able to identify all regions that are associated with the occurrence of a given term. Please note, pReverse refers to the p-value of the Bayesian reverse inference decoding determining the probability of the presence of a label term, given selection of that label and the prior probability of having that label.

At this point of the analysis, the reason for the occurrence of a term might be threefold as follows:

1. The term occurs mainly in studies that report thalamic activation.
2. The term occurs mainly in studies that reported activation of the functionally connected region.
3. The term occurs in studies that reported activation in both the thalamus and the functionally connected region.

Please note that both the first and the third scenario would support thalamic involvement in the term, while scenario 2 would not support thalamic involvement. To exclude an above-chance influence of the second scenario, we repeated the decoding for all the functionally connected HCP-MMP regions, without the mask from the respective thalamic cluster (see Fig. 1B). We then used the resulting region–term associations as a quasi-null-model to normalize the results. Having created another dataset (based on the same steps as for the terms of the thalamic-cortical decoding), we calculated the *intersection* between the set of terms from the thalamo-cortical decoding and the set of terms from the decoding of the functionally connected regions with a *logical-&-conjunction *(see Fig. 1C*)*. This step of the analysis returned all common terms between the set of terms from the thalamo-cortical and the cortical-only decoding with no repetitions.

In the next step of the analysis, we inspected all regional IDs with regard to the terms they were tagged with. More specifically, we calculated the *set difference* between regional IDs of thalamic-cortical terms and the terms from the cortical decoding (see Fig. 1D). This returned all terms tagged with an ID that were not present in the set of terms of the cortical decoding, without repetitions. With the completion of these steps, we were able to generate reverse inference maps for all thalamic-cortical pairs and unraveled all regions within that “system” that were commonly associated with a given term (see Fig. 1E). Of note, while decoding was performed with the NeurosynthDecoder, the subsequent analysis of the terms was performed with an in-house MATLAB (MATLAB 2021) script.

For every thalamic-cortical system, we then calculated the percentage of regions that were associated with a specific term, compared to the total number of region–term associations within that functionally connected system. To report a measurement of effect size, we also calculated the average Bayes Factor of all terms that build a putative functional brain system. Bayes Factor was calculated as the ratio between the posterior odds and the prior odds (ref., Goodman 1999; Poldrack 2006). Multiple comparison was applied separately for thalamo-cortical pair (and in case of the quasi-null-model for each cortical region).

This resulted in estimates of the overall association strength of a given term with the respective thalamo-cortical system. To aid and validate our interpretation, we aggregated the terms of the systems-level decoding into larger topics, as provided by Neurosynth (Yarkoni et al. 2011). The topics were originally derived from text-mining abstracts of published neuroimaging papers through latent Dirichlet allocation (LDA) (for details see (Poldrack et al. 2012; Rubin et al. 2017; de la Vega et al. 2018). We used the most recent version with 50 topics, derived from the abstracts of 14.371 articles in the Neurosynth database. A detailed description of how we aggregated the terms into larger topics can be found in the supplementary material (supplementary Figure S6).

As a final step, we created surface-based maps for every significant, unique term of each thalamic-cortical system to display the systems that are associated with the processing of a given psychological construct. The visualization was done with the connectome workbench commands (Marcus et al. 2011) and in-house MATLAB (MATLAB 2021) scripts. A possible caveat of this approach is the use of task-constrained meta-analytic co-activation maps for delineating thalamic clusters while using RSFC analysis to identify functionally coupled cortical areas. We therefore repeated the whole decoding with BrainMap-based meta-analytic coactivation maps for each thalamic cluster. A detailed description of this complementary validation, including a comparison of the results between both analyses, can be found in the supplementary material (S7–S12). There is compelling evidence that meta-analytic task-constrained co-activation mapping (i.e., MACM) and RSFC analysis can recover highly similar brain-wide systems (ref. Smith et al. 2009; Heckner et al. 2021). In the present research, we nevertheless decided to constrain the selection of cortical regions for the main analysis of the systems-level decoding by RSFC obtained from an independent data set to exploit its higher statistical power in detecting functionally connected cortical regions.

### Supplementary analysis

The rather strict systems-level decoding reduces the range of terms that are related to thalamic-cortical activation. That is, when we calculated the *set intersection* between the terms from the thalamo-cortical and the cortical-only decoding (i.e., the quasi-null-model), we also filtered out terms that are exclusively related to thalamic-cortical pairs, because this approach retains only the common elements of both sets. To increase the range of terms associated with thalamic-cortical activation, we subsequently calculated the *set difference* between the terms from both datasets. This represents a supplementary analysis with a more lenient thresholding and returned a set of all terms from the thalamic-cortical decoding that were not present in the set of terms from the cortical decoding with no repetitions. Since we could not unequivocally rule out whether this set of terms equally arose from activity within cortical regions, we did not include these results in the main description of thalamic-cortical brain systems.

## Results

### Co-activation-based parcellation

**Fig. 2:**
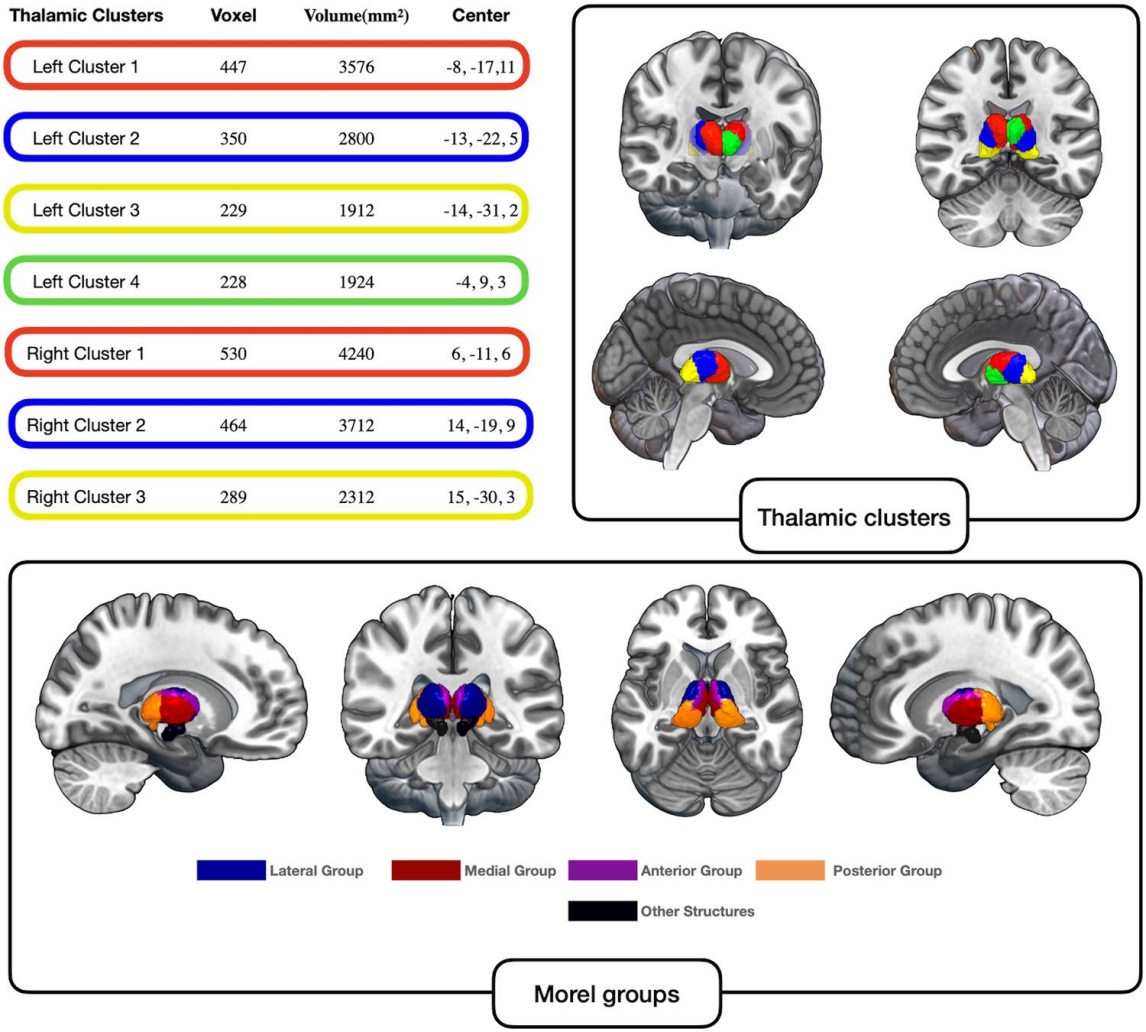
Voxel size, Volume and Center of Mass of the MACM-CBP-derived thalamic clusters. Colored frames around the table entries on the left correspond to the respective colors of the thalamic clusters displayed in the cortical renderings on the right. Because we report the overlaps of the task-based parcellation with the anatomical Morel atlas, we displayed also the major thalamic groups from the Morel atlas for sake of visual comparison. A table informing about the specific thalamic nuclei and a figure showing the axial slices of the nuclei, that form a given thalamic group is presented in the supplementary material (S13, S14). The cortical renderings were created with MRIcroGL (Rorden and Brett 2000)

We used MACM-CBP to create a novel data-driven parcellation of the human thalamus based on functional connectivity across task data. K-means clustering revealed the 4-cluster solution as the most stable clustering for the left thalamus. For the right thalamus, the 3-cluster solution was identified as the most stable one. Figure 2 depicts renderings of the optimal cluster solutions and provides additional information on the size and center of mass of each cluster of the optimal solutions. The MNI volumes of the thalamic subdivisions will be made publicly available in the ANMIA database (https://anima.fz-juelich.de/) upon publication.

To compare our parcellations based on task-constrained co-activations with existing parcellations based on histological information, we computed overlaps (i.e., Jaccard index) of each thalamic cluster with single nuclei of the canonical Morel atlas (Morel et al. 1997) (see Fig. 2 and supplementary Table S13 and supplementary Fig. 14 for details). The resulting overlaps were in accordance with the classical view of thalamic demarcation.

Of the selected 4 clusters for the left thalamus, cluster 1 covered the midline of the thalamus including medial (central lateral nucleus (CL), mediodorsal nucleus (MD)) and lateral (ventral lateral nucleus (VL)) nuclei; cluster 2 covered medial nuclei (central medial nucleus (CM), posterior nuclei (lateral posterior nucleus (LP), medial pulvinar (PuM)), and lateral regions (ventral lateral nucleus (VL); cluster 3 consisted mainly of the medial pulvinar (PuM), and cluster 4 covered medial (MD) and lateral nuclei (ventral anterior nucleus (VA)).

Of the 3-cluster solution found for the right thalamus, cluster 1 encompassed nuclei from the medial group (central lateral nucleus (CL), MD) and VA in the lateral division; cluster 2 covered as well medial nuclei (CL), a posterior nucleus (LP), and lateral regions (VL, VPL); and cluster 3 comprised the PuM.

### Resting-state functional connectivity

Seed-connectivity maps of each thalamic cluster were overlaid with the Glasser et al. (2016) HCP-MMP regions to generate cortical regions for the systems-level decoding approach. The 360 HCP-MMP regions (i.e., 180 per hemisphere) can be grouped into 22 larger cortices per hemisphere (see supplement of Glasser et al. 2016) that can be further subdivided into five cortical regions (i.e., anterior cortices, posterior cortices, early and intermediate visual cortex, sensorimotor areas, and auditory regions). We describe each seed-connectivity map based on spatial overlap with the 22 cortices.

**Fig. 3:**
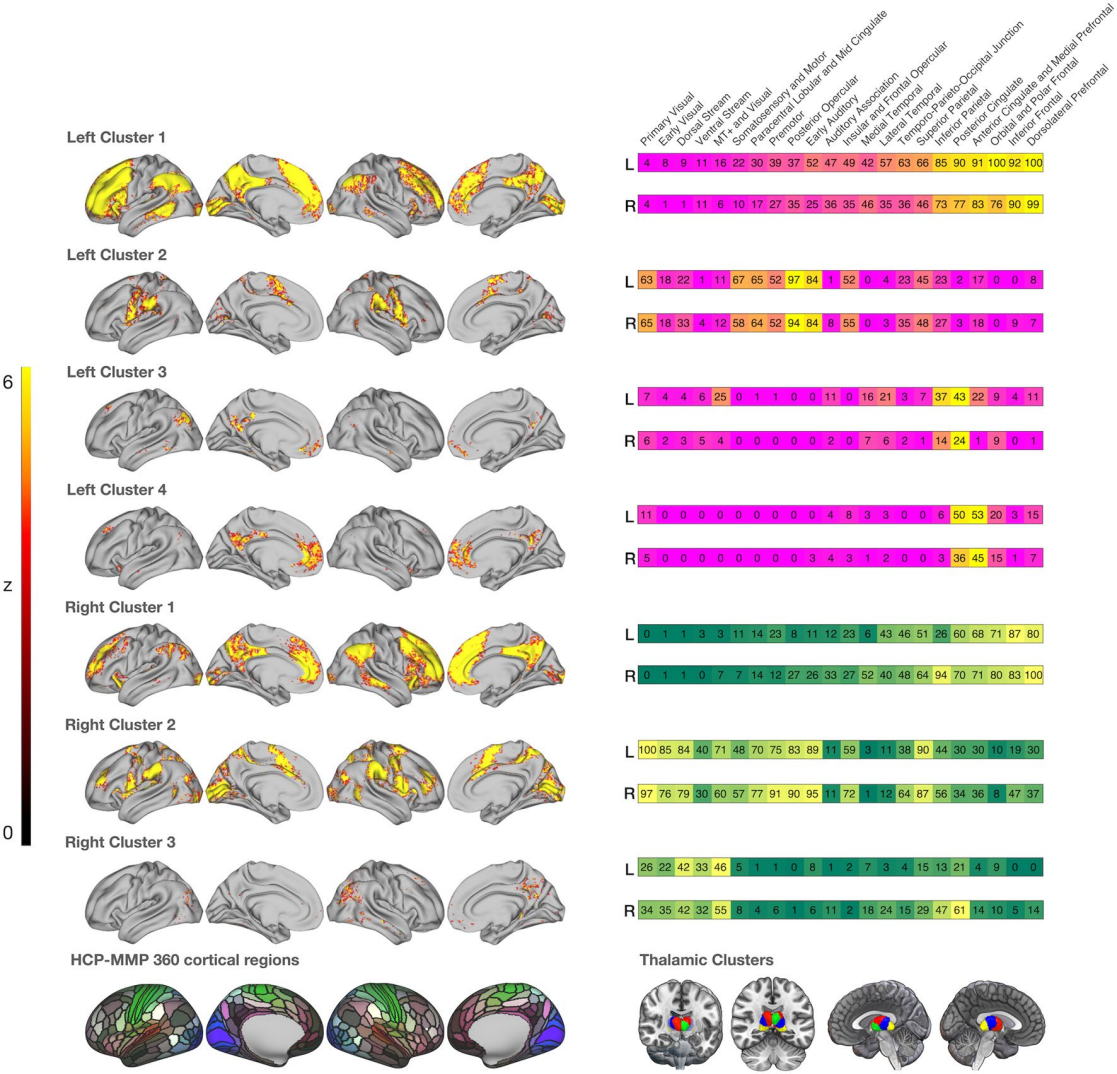
Surface maps created with connectome workbench commands (Marcus et al. 2011) displaying resting-state functional connectivity of the thalamic clusters with cortical regions. Heatmaps on the right display the overlaps of the cortical regions with the 22 cortices of Glasser et al. (2016). At the left bottom, we display the 360 HCP-MMP cortical regions the seed-connectivity maps were overlaid with. At the right, we present the renderings of the 4 (i.e., left hemisphere) and 3 (i.e., right hemisphere) thalamic clusters. Spatial overlap was quantified via the Jaccard index, and visualization was done with in-house MATLAB (MATLAB 2021) scripts

For left thalamic cluster 1, we found a total of 128 functionally connected HCP-MMP regions, located in large portions of the anterior regions of the cortex, in posterior regions of the cortex, in auditory and temporal regions and to a smaller extent in sensorimotor areas (for details see Fig. 3). Left cluster 2 was functionally connected with 47 regions encompassing large portions of sensorimotor areas, auditory regions (i.e., early auditory), and visual areas (i.e., primary visual), as well as smaller portions of the posterior regions of the cortex. For left cluster 3, we found seven functionally connected cortical regions in posterior cortex (i.e., posterior cingulate, inferior parietal), in left temporal regions and—to a smaller extent—in visual and anterior (i.e., anterior cingulate) regions of the cortex. Left cluster 4 was functionally connected with 16 regions located in bilateral anterior regions of the cortex, in the posterior (cingulate) cortex and to a small extent in the primary visual cortex.

Right cluster 1 was connected with a total of 97 HCP-MMP regions located in anterior and posterior regions of the cortex, in temporal and auditory regions and in smaller portions of sensorimotor areas. A total of 116 regions were functionally connected with right cluster 2. These were located in early and intermediate visual areas, auditory regions, sensorimotor areas and in anterior and posterior regions of the cortex. Finally, right cluster 3 was functionally connected with 15 regions, encompassing early and intermediate visual and posterior regions of the cortex and to a smaller extent regions in auditory, temporal, and anterior divisions of the cortex. The RSFC analysis provides further evidence for a separation of the thalamic clusters: Of the 226 cortical regions that were functionally connected to any of the seven seed regions, only 11 regions were linked to four (out of seven) seed regions, 34 to three, 99 to two, and 82 to one seed region.

### Functional characterization of thalamic clusters

We computed Bayesian reverse posterior probabilities (i.e., here denoted as probReverse) of functional terms obtained from the Neurosynth database for all thalamic clusters. This resulted in estimates for the probability of the occurrence of a term in the database, given the activation foci within a thalamic cluster. Left cluster 1 was related with ‘incentive delay’ (pReverse = 0.0025; probReverse = 0.726; Bayes Factor = 2.65) and ‘reward anticipation’ (pReverse = 0.007; probReverse = 0.706; Bayes Factor = 2.4), ‘nociceptive’ (pReverse = 1.123E−06 probReverse = 0.772; Bayes Factor = 3.39) and ‘autobiographical memory’ (pReverse = 0.00615; probReverse = 0.713; Bayes Factor = 2.48). Left cluster 2 was associated with sensorimotor processes (i.e., ‘finger tapping’ (pReverse = 3.90E−10; probReverse = 0.792; Bayes Factor = 3.53) and ‘motor task’ (pReverse = 4.77E−09; probReverse = 0.759; Bayes Factor = 3.15). Left cluster 3 was related to the processing of word-pairs (pReverse = 0.008; probReverse = 0.799; Bayes Factor = 3.98). Left cluster 4 was involved with ‘incentive delay’ (pReverse = 0.0055; probReverse = 0.751; Bayes Factor = 3.02), the processing of ‘sexual’ stimuli (pReverse = 0.00015; probReverse = 0.775; Bayes Factor = 3.44) and ‘chronic pain’ (pReverse = 0.008 probReverse = 0.738; Bayes Factor = 2.82). The results for the decoding of the right hemisphere showed commonalities but were at the same time less concise. That is, right cluster 1 was related to ‘incentive delay’ (pReverse = 0.00021; probReverse = 0.732; Bayes Factor = 2.73)), right cluster 2 to the perception of noxious stimuli (i.e., ‘noxious’ (pReverse = 8.28E−10; probReverse = 0.775; Bayes Factor = 3.44), ‘nociceptive’ (pReverse = 9.02E−05; probReverse = 0.743; Bayes Factor = 2.89), and for right cluster 3 no term related to cognitive or perceptual processes surpassed the significance threshold. Please note that we excluded anatomical and technical terms from the description of the results since we were mainly interested in psychological constructs. All terms denoting psychological constructs are displayed in Fig. 5, whereas a list of all top10 terms is given in the supplementary material (supplementary Table S15).

### Systems-level decoding of thalamic clusters

The present research aimed to broaden the perspective on the functional repertoire of distinct thalamic clusters. Since cognitive processes emerge from the interaction of interconnected brain regions, we decided to apply a systems perspective on thalamocortical functional decoding.

**Fig. 4:**
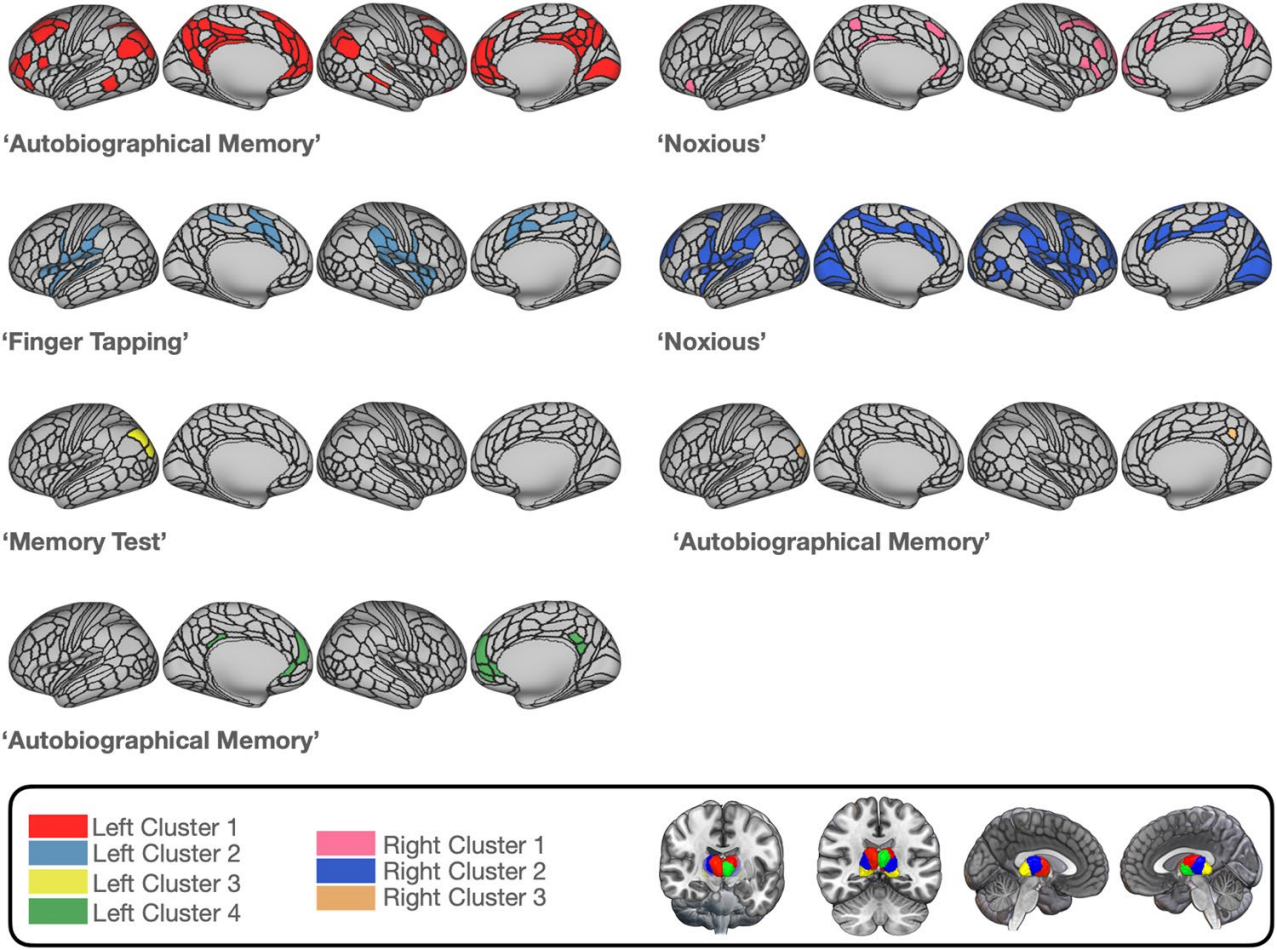
Surface maps of the largest systems per thalamic cluster with an outline grid representing the 360 HCP-MMP regions. The largest system is defined as the thalamic-centered system with the highest percentage of co-activated cortical regions associated with a given term. Color gradings of the surface maps correspond to each thalamic cluster represented in the box on the bottom-left corner of the figure

**Fig. 5:**
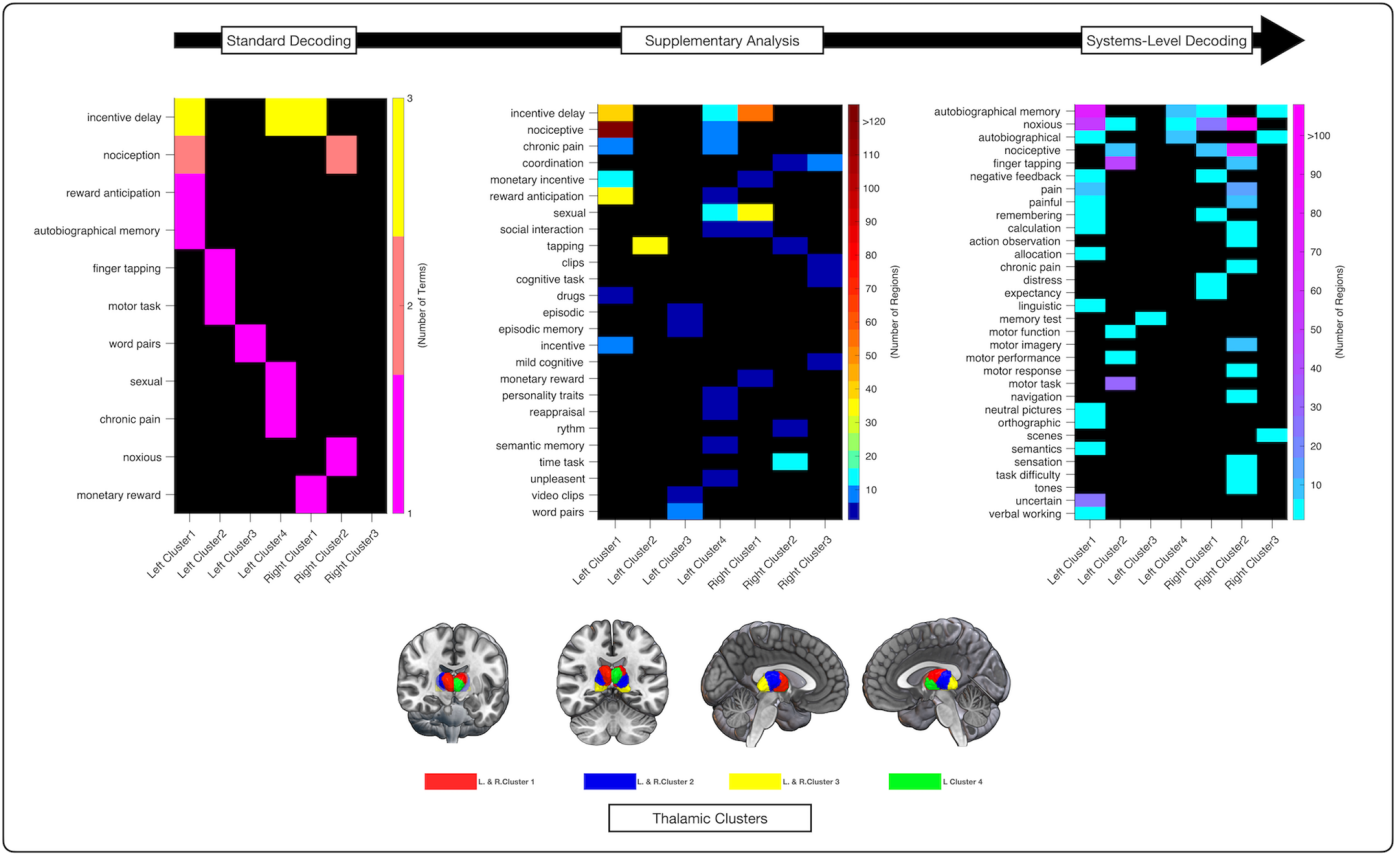
Terms of the different decoding steps for the left and right thalamic clusters summarized as heatmaps. While the visualization of the terms for all decoding steps is hierarchically organized by the number of term appearances, the color-graded heatmaps for the supplementary Analysis and the systems-level decoding reflect the number of cortical regions co-activated with a given thalamic cluster. Down below we inserted the respective renderings of the thalamic clusters, for ease of readability. Visualization of the heatmaps was done with an in-house MATLAB (MATLAB 2021) script

*Left cluster 1.* For left cluster 1, the system decoding resulted in a total number of 39 distinct terms, covering psychological constructs, anatomical and as well technical terms. The terms denoting psychological constructs are illustrated in Fig. 5. The term ‘autobiographical memory’ had the largest percentage of regional associations (i.e., 72/123*100 =  ~ 59%), followed by “noxious” (~ 40%) and ‘uncertain’ (~ 17%). Other terms denoting psychological constructs such as ‘verbal working’, ‘remembering’, ‘linguistic’, and ‘calculation’ were only associated with a few thalamo-cortical couples with percentages below 5%. A complete list of all terms, their percentages, the number of regions, and the mean Bayes Factor per system for each thalamic cluster, can be found in the supplementary material (supplementary Table S16). The ‘autobiographical memory’- system covered regions in the bilateral posterior and anterior division of the cortex as well as the right primary visual cortex as displayed in Fig. 4.

*Left cluster 2.* Thalamic-cortical couples for left cluster 2 produced mainly terms from the spectrum of sensorimotor processes. Roughly 95% (i.e., 45/47*100) of all thalamic-cortical couples produced the term ‘finger tapping’, closely followed by ‘index finger’ (~ 70%) and ‘motor task’ (~ 66%). The ‘finger tapping’-system covered large parts of sensorimotor areas, auditory regions and—to a smaller extent—anterior, posterior, and visual regions of the cortex.

*Left cluster 3*. For thalamic-cortical regions of left cluster 3, the system decoding procedure yielded only the single term ‘memory test’. The thalamic-cortical couple involved with the term ‘memory test’ was located in the left inferior parietal cortex.

*Left cluster 4*. Five terms were associated with activation in thalamic-cortical couples for left cluster 4. The largest percentage was found in an ‘autobiographical memory’ system (i.e., 9/15*100 = 60%), followed by the term ‘autobiographical’ (33.3%) and ‘noxious’ (6.6%). Associations for the ‘autobiographical/autobiographical memory’-system were located in bilateral anterior (i.e., ACC and medial prefrontal) and posterior (i.e., PCC) divisions of the cortex.

*Right cluster 1.* 16 terms were associated with thalamic-cortical couples of the right cluster 1. The largest percentage was found for the term ‘noxious’ (i.e., 25/97*100 =  ~ 26%). Other terms denoting psychological constructs showed low association strengths (e.g., ‘autobiographical memory’, ‘remembering’ =  ~ 1%). The “noxious” system encompassed mainly anterior and posterior divisions of the cortex and small portions of the right premotor cortex and left insular and frontal opercular cortex.

*Right cluster 2.* The reverse inference decoding of the thalamic-cortical couples for cluster 2 produced 62 distinct terms in total. The largest percentage was found for the term ‘noxious’ (i.e.,108/116*100 =  ~ 93%) and ‘nociceptive’ (~ 73%), followed by ‘finger movements’ (~ 21%). Other terms denoting psychological constructs such as ‘action observation’ (~ 1.7%), ‘calculation’ (~ 0.8%), ‘chronic pain’ (~ 2.6%), ‘motor imagery’ (~ 3.4%), ‘navigation’ (~ 2,6%) and ‘pain’ (~ 8.6%) were less pronounced in their region–term associations. The ‘noxious’ system for cluster 2 showed strong overlaps with the ‘noxious’ system of cluster 1. As a major difference, we here found a large portion of region–term associations in visual and auditory areas that were not a part of the functional system of right cluster 1.

*Right Cluster 3.* Three terms were associated with activation in the thalamic-cortical couples of right cluster 3. The largest percentage was found for the term ‘autobiographical’ (i.e., 2/3*100 = 66.6%), followed by ‘autobiographical memory’ (33,3%) and ‘scenes’ (33.3%). The ‘autobiographical/autobiographical memory’ system covered small portions of the left inferior parietal cortex and the right PCC.

*Neurosynth topics.* For three topics, we found a rich repertoire of terms from the spectrum of somatosensory functions (i.e., Topic 17), pain and nociception (Topic 32), and memory (i.e., Topic 33), which are associated with the activation of most of the thalamic clusters (see Supplementary Figure S7). Moreover, we also found a large number of topics with only few associated terms and reported activations within single thalamic clusters. These results represent a good validation of the systems-level decoding, as the distribution of terms in the topics are also reflected in the percentages of cortical regions coactivated with the thalamus and the spatial extent of the associated thalamocortical brain systems (i.e., the larger the system, the more consistent terms fall into a particular topic and vice versa).

### Supplementary analysis

In a supplementary analysis, we applied a more lenient thresholding to the systems decoding because its standard implementation described above, solely focused on the terms exclusively associated with the thalamic-cortical regions, excluding all terms associated only with cortical regions. As expected, the supplementary analysis yielded a more diverse spectrum of terms denoting psychological constructs for most of the thalamic clusters (see Fig. 5). Terms of thalamic-cortical pairs of left cluster 1 included ‘chronic pain’, ‘drugs’ and reward processes (i.e., ‘incentive’, ‘incentive delay’, ‘monetary incentive’, ‘reward anticipation’). The calculation of the *set difference* between the terms of thalamic-cortical pairs and the quasi-null-model for left cluster 2 added no relevant information (i.e., denoting no additional psychological constructs). For left cluster 3, the procedure added two additional terms: ‘episodic memory’ and ‘word pairs.’ Thalamic-cortical pairs of left cluster 4 were related to ‘chronic pain’, ‘incentive delay’, ‘reward anticipation’, ‘semantic memory’, ‘sexual’,’ reappraisal’, ‘personality traits’ and ‘social interaction’.

Terms of right cluster 1 that did not show an intersection with terms linked to cortical regions included *‘*incentive delay’, ‘monetary incentive’, ‘monetary reward’, ‘sexual’ and ‘social interaction’. For right cluster 2, the procedure yielded the terms *‘*coordination’, ‘rhythm’, and ‘time task’*.* Finally, the calculation of the *set difference* between the terms of thalamic-cortical couples of right cluster 3 and of only cortical regions produced the terms ‘coordination’, ‘cognitive task’ and ‘mild cognitive’. A list of all terms obtained in the supplementary analysis is provided in supplementary Table S17.

## Discussion

We aimed at the functional characterization of thalamic parcels through meta-analytic decoding across the cognitive neuroscience literature. We created a novel parcellation of the thalamus, optimized for this purpose. We utilized two complementary decoding strategies: the standard Bayesian reverse inference decoding, as described in the literature (Poldrack 2006; Eickhoff et al. 2011), and a novel systems-level decoding approach, which we propose for the joint decoding of target regions with functionally connected cortical regions. The systems-level decoding revealed co-activations of thalamocortical pairs, constrained by the context of different cognitive and behavioral tasks. With the novel systems decoding approach, we were able to detect thalamus-centered cognitive cortical systems that are engaged in sensorimotor processes, nociception, and autobiographical memory.

### A novel task-dependent parcellation of the left and right thalami

We propose a novel parcellation of the thalamus with four left and three right thalamic subunits based on whole-brain co-activation patterns as revealed by MACM-CBP, optimized for subsequent functional decoding. The clustering solutions with four and three subunits, respectively, showed the highest stability across topological, information-theoretic, and cluster separation metrics.

With four and three clusters per hemisphere, our clustering solution features fewer and larger clusters than previously proposed solutions from structural or functional connectivity data, which distinguish either 7 (Behrens et al. 2003; Battistella et al. 2017; Najdenovska et al. 2018), 8 (Fan et al. 2016), 9 (Hwang et al. 2017), 11 (Saranathan et al. 2021), 15 (Kumar et al. 2017) or 21 (Kumar et al. 2015) distinct subunits per hemisphere. Given this discrepancy, it is important to emphasize that our clustering corresponds well with established cytoarchitectural properties of the thalamus, as described by the Morel atlas (Krauth et al. 2010; Morel et al. 1997). Cluster 1 corresponds with medial and lateral nuclei, cluster 2 corresponds with medial, posterior, and ventral-lateral nuclei, cluster 3 corresponds with the pulvinar, and left cluster 4 comprises medial and ventral anterior nuclei. Cytoarchitectonically defined nuclei are commonly grouped according to their afferent projections (Sherman and Guillery 2013; Sherman 2016): First order nuclei such as the ventral posterior (VP), medial (MGN), and lateral geniculate nuclei (LGN) receive input from sensory organs and other subcortical structures and relay this information to the cortex (Sherman and Guillery 2013; Sherman 2016). Higher order nuclei such as the AV, MD, pulvinar, and CM, forward information from one cortical region to another via cortico-thalamo-cortical pathways (Sherman et al. 2006). A different proposal classifies thalamic nuclei into three groups: (a) MGN, LGN, VPL, VL, and VA as principal sensorimotor nuclei, (b) lateral MD, lateral dorsal nucleus (LD), and LP as sensorimotor/limbic nuclei, and (3) AT, medial MD, CM, and the smaller midline intralaminar nuclei (IL) as limbic nuclei (Vertes et al. 2015). We were able to find a similar classification of nuclei in this study, however, with the exception that limbic and limbic/sensorimotor were merged into the same clusters. While cluster 2 (ventral-lateral nuclei) fit the description of principal sensorimotor nuclei, cluster 1 (medial and lateral nuclei) comprised not only limbic but also limbic/sensorimotor nuclei.

MACM-CBP was thus able to detect anatomically meaningful thalamic subregions which is in line with previous MACM-CBP studies on other brain regions that were also able to capture subunits that correspond with anatomical boundaries (Bzdok et al. 2013; Caspers et al. 2014; Ray et al. 2015). Since MACM-CBP uses task co-activation patterns as input data, the subregions derived from it reflect the functional resolution of task fMRI studies, which is the key prerequisite for subsequent functional decoding. We would also like to emphasize that fMRI preprocessing parameters such as smoothing kernels and the comparatively low spatial resolution of the larger corpus of past fMRI studies would make it unlikely to delineate smaller clusters. Functional imaging studies have only recently achieved appropriate resolution to detect dissociable signals in larger thalamic subnuclei (Kumar et al. 2017; Hwang et al. 2017; Iglesias et al. 2018), while smaller nuclei are still out of reach with current scanning technology (Antonucci et al. 2021).

### Functional characterization of thalamic clusters

As a relay, the thalamus is responsible for transmitting multimodal sensory information to the cortex (Sherman et al. 2006), particularly through the ventral nuclei. The decoding resulted in terms related to sensorimotor processes (i.e., ‘finger tapping’, ‘finger movements’, ‘motor task’) for left and right thalamic cluster 2, which most prominently includes the ventral nuclei. A crucial part of the sensorimotor information that is transmitted, integrated, and modulated by thalamic nuclei concerns nociceptive information (Sotgiu 2001; Yen and Lu 2013). The decoding revealed associations between nociception/pain processing and left/right cluster 1, left cluster 4, and right cluster 2, which include medial, central, and (ventral) lateral nuclei of the thalamus. Pain-related associations with clusters that encompassed ventral lateral nuclei—which are typical relays—but also with medial nuclei that represent higher order thalamic nuclei might be a consequence of the different components of nociceptive signals which are transmitted by different fibers within the spinothalamic tract: The spinothalamic tract, through which pain-related information is conveyed to the cortex, projects to lateral ventral posterior and to medial (e.g., intralaminar) nuclei of the thalamus (Lenz et al. 2004). Different aspects of experiencing noxious sensations include a sensory-discriminative component mediated by lateral spinothalamic tract projecting to somatosensory cortices and an affective-motivational component represented by medial spinothalamic tract projecting to cortical areas (e.g., ACC and prefrontal cortex) (Apkarian et al. 2005, 2011). Next to sensorimotor associations, we were also able to link memory functions to the thalamus: Left cluster 1 (including MD) was associated with ‘autobiographical memory,’ and left cluster 3 (consisting of PuM) with ‘word pairs.’ Autobiographical memory is based on episodic retrieval but also depends on emotional, attentional, and executive functions (Conway and Pleydell-Pearce 2000; Svoboda et al. 2006; Burianova and Grady 2007). The observed association with autobiographical memory fits with converging evidence from clinical neuropsychology, animal research, and modern neuroimaging that has linked the MD to mnemonic processes, including episodic memory (Aggleton and Brown 1999; Harding 2000; Pergola et al. 2012, 2013; Mitchell 2015; Wolff et al. 2015; Wolff and Vann 2019). Word pairs, on the other hand, are related to verbal associative memory (Arndt 2012). The observed association between word pairs and the thalamic cluster that includes the pulvinar is less straightforward, as the previous literature has mostly discussed the pulvinar with respect to its relationship to visuospatial attention, visual processing, and the integration of visual information (Shipp 2003; Arend et al. 2008; Saalmann and Kastner 2011)). Some work, however, suggests a role of the pulvinar in the visual processing of mnemonic information (Rotshtein et al. 2011; Kafkas et al. 2020) which could explain the reported association with word-pairs that are usually presented visually.

Finally, left/right cluster 1 and left cluster 4 were associated with reward-related terms (i.e., ‘incentive delay’, ‘reward anticipation). All three clusters encompassed portions of MD, which is known to forward information from striatal to cortical regions, especially in the anticipation of a reward as part of the cortico-striato-thalamo-cortical reward circuit (Knutson and Greer 2008; Haber and Knutson 2010; Cho et al. 2013; Huang et al. 2018; Oldham et al. 2018).

While the standard decoding approach revealed meaningful associations with respect to the literature, the general yield of this method was surprisingly low, given the particular importance of the thalamus for brain function. As it is easily conceivable that most of the behavioral repertoire of the thalamus only arises from its interactions with the cerebral cortex, a joint decoding of thalamic clusters with cortical regions would likely be more appropriate. While characterizing the segregation and specialization of brain areas in regard to behavioral functions is a hallmark of cognitive neuroscience (cf., (Genon et al. 2018a)) it has become increasingly apparent that motor, sensory, and higher cognitive functions draw most heavily upon interactions between brain areas and their integration into functional systems (Rubinov and Sporns 2010; van den Heuvel and Sporns 2013; Sporns and Betzel 2016). The thalamus is a central hub region within the brain network and participates in several brain-wide systems through its reciprocal interactions with cortical regions via thalamocortical loops (Jones 2001; Sherman and Guillery 2013; Halassa and Sherman 2019). The characterization of the thalamus’ functional repertoire should therefore benefit from a joint perspective on thalamic subregions and the neocortex. Thus, building on previous efforts to decode entire subsystems involved in cognitive control (Langner et al. 2018), we applied a novel decoding strategy and investigated behavioral associations of thalamic clusters in conjunction with functionally connected cortical areas.

### Functional characterization of thalamocortical systems

The systems decoding revealed additional terms related to language (i.e., ‘linguistic, ‘orthographic’, ‘semantics’), memory processes (‘verbal working’, ‘remembering’), and locomotion (‘navigation’). Language-related terms were all associated with thalamo-cortical pairs of left cluster 1 (medial–lateral nuclei). While thalamic participation in language is widely described in the neuropsychological literature (Brown 1974; Crosson 1984, 2021; De Witte et al. 2011), the evidence from functional neuroimaging is limited (Klosterman et al. 2013; Llano 2013, 2016). When imaging studies have reported involvement of the thalamus in language processes, they either feature very broad anatomical labels (e.g., “thalamus”) for reporting activation peaks or utilize anatomical regions of interest that do not discriminate between thalamic nuclei, which limits the available evidence to the issue of hemispheric specialization (Indefrey and Levelt 2004; Garn et al. 2009; Seghier and Price 2010). Functional connectivity studies, however, have prioritized connections between cortical regions in inferior frontal and superior temporal cortex and the thalamus in language (Llano 2013, 2016), which supports the here observed co-activations between these cortical regions and left thalamic cluster 1 during language tasks. Based on our systems decoding, we conclude that the thalamic part in these connections is likely to include the medial nuclei that are prominently featured in left cluster 1 and suggest this subregion as a viable starting point for future studies on language functions of the thalamus.

The thalamus’ involvement in memory functions is best documented for working memory, where the thalamus is thought to participate in the executive part of working memory through striato-thalamo-prefrontal loops (D’Esposito and Postle 2015). Cells in the MD show sustained firing during the delay period of working memory, similar to neurons in PFC (Fuster and Alexander 1971), and most work so far has focused on the interaction between MD and dorsolateral PFC for working memory (Funahashi 2013; Wolff and Vann 2019). While our systems decoding implicated the left thalamic cluster 1, which includes MD, in working memory, we observed this association only in conjunction with more posterior frontal parts in left premotor cortex. Premotor cortex itself seems to play a role in working memory, depending on stimulus content and its level of abstraction (Christophel et al. 2017; Badre and Nee 2018). The here reported association can thus be regarded as a promising first lead for more focused investigations of thalamo-frontal connectivity in working memory.

To the best of our best knowledge, in the human neuroimaging literature only few studies have found the thalamus to be involved in navigation and locomotion embedded in subcortical-cortical systems (Jahn et al. 2004; Ionta et al. 2009; Hao et al. 2016); and if so, without discriminating between thalamic nuclei. In contrast, findings from animal research paint a more nuanced picture, pointing towards an involvement of medial and anterior nuclei in spatial navigation (Cain et al. 2006; Jankowski et al. 2013; Ito et al. 2015) and of the LGN, the reticular and the ventrolateral nuclei in locomotion (Marlinski et al. 2012b; Aydın et al. 2018; Beloozerova and Marlinski 2020). We found an association between central-ventral-lateral right cluster 2 in conjunction with somatosensory regions, visual regions, and superior parietal cortex and the term ‘navigation*’.* According to a model derived from research on the cat brain, motor and sensory information converge in the ventral lateral nuclei, while these signals are transmitted via the VL to the motor cortex during locomotion (Marlinski et al. 2012a). Our systems decoding results*,* however, highlight the need for further research in humans to characterize the contribution of the human thalamic nuclei in locomotion*.*

The more liberal supplementary analysis resulted in further terms: ‘personality traits’ and ‘social interactions’ (right cluster 1), ‘mild cognitive’ (right cluster 3), and ‘semantic memory’ (left cluster 4). More work is needed to elucidate which personality traits and what kind of social interactions are supported by thalamic-cortical interactions. Animal studies have linked MD–prefrontal cortex circuits to social dominance (Zhou et al. 2017), which could point towards social personality characteristics such as extraversion as promising candidates. The associations with ‘semantic memory’ and ‘mild cognitive’ (as in mild cognitive impairment) again highlight the role of the thalamus in memory processes, which we will address next.

### Thalamocortical systems

For some behavioral terms, the systems-level decoding revealed widespread associations, pointing at several cortical regions that were co-activated with the thalamus in studies on the given term. The probabilistic relationship between the occurrence of the behavioral term and thalamocortical co-activations suggests the involvement of thalamocortical systems in a given behavior. The joint involvement of the ventral posterior nuclei (i.e., first order nuclei) of the thalamus with cortical regions in somatosensory, motor, premotor, auditory, and visual cortex in various sensorimotor processes, such as ‘finger tapping’, ‘motor task’), is well described in the literature (Sherman et al. 2006; Sherman 2016; Zhang and Li 2017). The fact that such a system was recovered by the novel systems-level decoding emphasizes the solid foundation of this approach and gives us confidence to discuss the less well-described and potentially clinically relevant thalamo-cortical systems involved in autobiographical memory and nociception in more detail.

### The autobiographical memory system

**Fig. 6:**
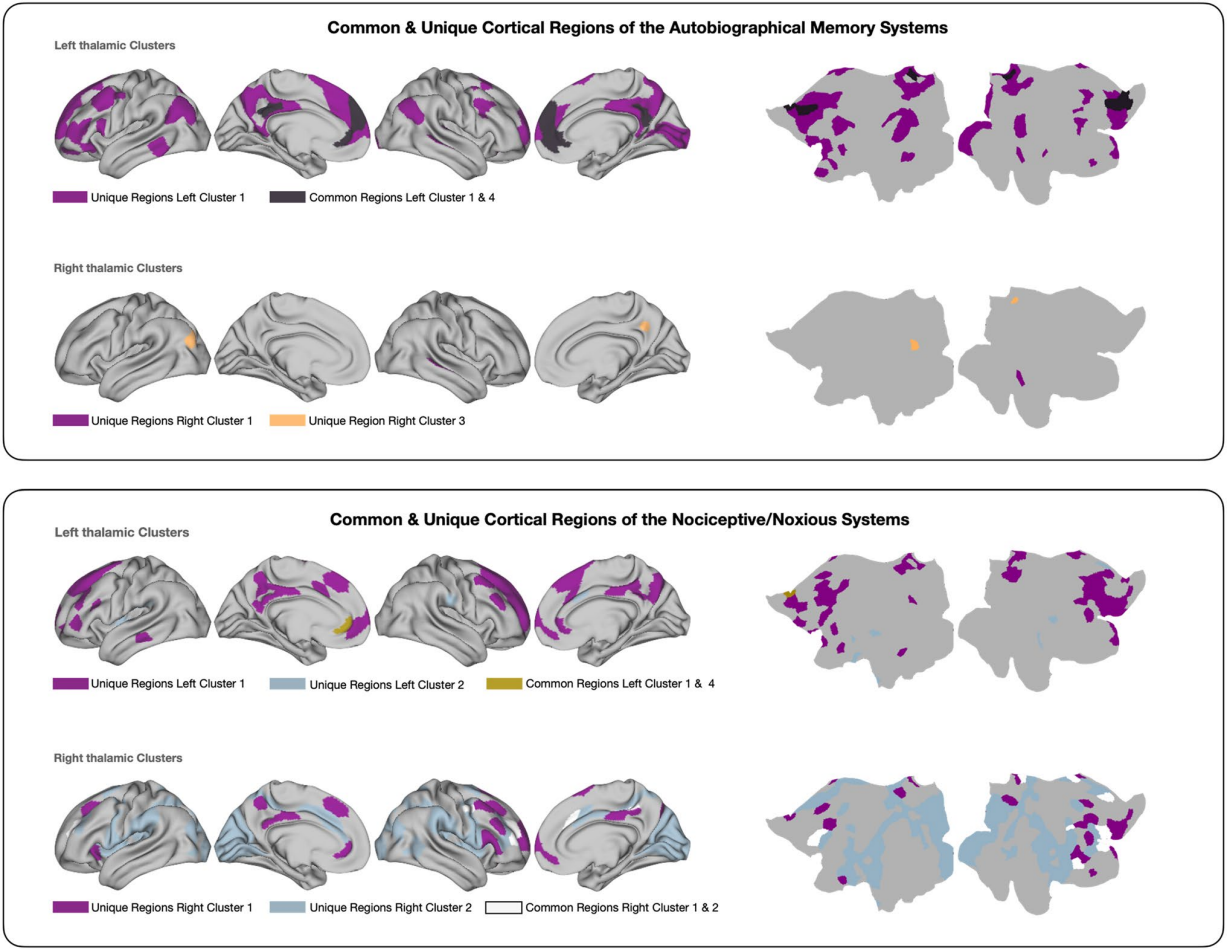
Surface and flatmaps of unique and common cortical regions of the Autobiographical Memory System and the Nociceptive/Noxious System. Unique regions represent cortical regions showing co-activations with a single MACM-CBP-derived thalamic cluster only, while co-activations between several thalamic clusters and cortical regions that overlap are displayed as common regions. Visualization was done with connectome workbench commands (Marcus et al. 2011)

In terms of spatial extent, the autobiographical memory system is mostly centered on left cluster 1, whereas left cluster 4, right cluster 1, and right cluster 3 contribute in conjunction with fewer cortical regions (see Fig. 6). Notably, the majority of the involved thalamic clusters contain mediodorsal and anterior thalamic subdivisions, which have been consistently linked to episodic memory. In addition, the pulvinar in right cluster 3 has been linked to attentional and visual aspects of memory processes. Overall, the thalamo-centered part of the autobiographical memory system covers bilateral prefrontal cortices, anterior and poster cingulate cortices, areas in inferior and superior parietal cortices, and primary visual areas all of which play distinct and collaborative roles in autobiographical memory (Spreng et al. 2009; Spreng and Grady 2010; Demblon et al. 2016; Monge et al. 2018).

Tremendous support for the involvement of the human MD and ventral anterior nuclei in memory circuits stems from the following clinical evidence: structural alterations in both thalamic nuclei have been associated with the memory impairments accompanying the alcohol-induced Korsakoff’s syndrome (Kopelman 1995; Harding 2000; Aggleton and O’Mara 2022) or after thalamic infarction (Schmahmann 2003; Pergola et al. 2012; Nishio et al. 2014; Danet et al. 2015). Furthermore, their functional role in mnemonic processes has been differentiated based on interconnections with subcortical and cortical brain areas in recollection and familiarity. In brief, recollection can be described as the retrieval of qualitative underpinnings of an episode (e.g., the contextual information), whereas familiarity reflects the strength of a memory (Yonelinas 2001, 2002; Yonelinas et al. 2010). Rodent and non-human primate research suggests that the anterior nucleus forms a node of a hippocampal-thalamo-prefrontal system involved in recollection memory, whereas the MD—through reciprocal connections with frontal areas such as PFC and ACC—is associated with familiarity (Aggleton and Brown 1999; Jankowski et al. 2013; Ketz et al. 2015; Wolff et al. 2015; Pergola et al. 2018; Aggleton and O’Mara 2022). Recent evidence from human imaging indicates an analogous dissociation between both thalamic nuclei and their connected (frontal) regions in recollection and familiarity-based memory processes (Pergola et al. 2013; Kafkas et al. 2020; Geier et al. 2020). The Autobiographical system in this study covered, however, parietal and visual areas in addition to the frontal ones, the dissociation between the retrieval of a memory (i.e., recollection) and the subjective feeling of familiarity is reflected in activation differences along the ventral (i.e., angular gyrus and temporoparietal junction) and dorsal (i.e., intraparietal sulcus) posterior parietal cortices (Yonelinas 2005; Berryhill et al. 2007; Johnson et al. 2013; Frithsen and Miller 2014). Evidence reporting equivalent dissociable (functional) connections between anterior nuclei and inferior parietal cortex and between the MD and the intraparietal sulcus in long-term memory (Spets and Slotnick 2020) indicates that thalamo-parietal connections might be equally important for differentiating recollection and familiarity aspects in episodic memory.

Altogether, there is only sparse evidence in the literature on a connection of parietal regions with the medial or ventral anterior thalamus in relation to episodic memory processes. To the best of our knowledge, findings on the conjunction of parietal and thalamic nuclei in memory are restricted to a circuit involving the posterior parietal cortex, PFC, and the medial pulvinar (Jutras et al. 2013; Park et al. 2014; Ketz et al. 2015; Homman‐Ludiye and Bourne 2019). Here, we report the PCC and the inferior parietal cortex as cortical components of the thalamo-centered part of the autobiographical memory system, centered on pulvinaric right cluster 3. The inferior parietal cortex and the medial pulvinar have been implicated in attentional and visual aspects of memory (Cabeza et al. 2008; Rotshtein et al. 2011; Homman‐Ludiye and Bourne 2019). We, therefore, suggest that frontal and parietal regions are jointly involved with the thalamus in attentional, executive, or even self-referential aspects of autobiographical memory processes. Remembering past events involves voluntary and involuntary memory retrieval (Cabeza and St Jacques 2007; Burianova and Grady 2007), search for and monitoring of memories (Corbetta et al. 2008; Vossel et al. 2014; Monge et al. 2018), and the self-referential reflection upon personal episodes (Spreng and Grady 2010; Demblon et al. 2016). Thus, autobiographical memory is phenomenologically complex and is likely to involve different brain networks. Along the same line, mapping of the thalamic nuclei with different intrinsic cortical networks including default-mode, task-positive dorsal and ventral frontoparietal, and the frontal executive networks showed coherence between studies examining the participation of the thalamus in cortical functioning (Fox et al. 2005; Yuan et al. 2016; Hwang et al. 2017).

In summary, scanning the literature revealed compelling evidence for thalamic involvement in episodic memory, confirming the results of our systems-level decoding. At the same time, the systems-level decoding represents a comprehensive account of the neuroimaging literature in which only in a few cases, thalamo-frontal and thalamo-parietal components of autobiographical memory processes have been simultaneously considered. Future work may want to build on the hypotheses generated here, to achieve a more complete representation of thalamic activity embedded in neural systems.

### The nociceptive system

The nociceptive system centered on thalamic clusters that include nuclei which are traditionally assigned to sensory processes (i.e., central-ventral-lateral left and right cluster 2). Additionally, the nociceptive system centered also on thalamic clusters consisting of higher order nuclei (i.e., medial and medial-anterior left clusters 1 and 4 and right cluster 1). Of note, the clusters comprising sensory nuclei and the clusters containing higher order nuclei exhibited dissociable co-activations with different sets of cortical regions (see Fig. 6), which might be an indicator for their different roles within the thalamo-centered part of the nociceptive system: Consensus views hold that nociceptive signals from the body are transmitted via two different spinothalamic pathways to cortical and subcortical regions (Lenz et al. 2004; Yen and Lu 2013; Groh et al. 2017). The lateral pathway ascends through the ventral posterior nuclei, relaying nociceptive information to somatosensory cortices, whereas the medial pathway propagates nociceptive information through midline and medial nuclei (e.g., intralaminar and MD) to limbic cortical areas. Fittingly, left and right clusters 2, which mainly consist of sensory relay nuclei, showed large task-constrained co-activations with somatosensory regions. Also, left, and right clusters that include medial higher order nuclei showed widespread co-activations with prefrontal regions and the cingulate. Joint involvement of prefrontal areas and the medial thalamus in nociception can have different implications: The ability to maintain painful stimuli in working memory is thought to be accomplished by connections between prefrontal cortex and the medial thalamus (Tseng et al. 2017). Spinothalamic projections to the ACC and the insula have been associated with the affective-motivational component of pain perception, which mediates avoidance behaviors and storage in long-term memory (Apkarian et al. 2005, 2011; Groh et al. 2017; Meda et al. 2019). The medial pain pathway is also concerned with orienting and other attentional processes (Bastuji et al. 2012). In line with this reasoning, two neuroimaging studies examining how attention is guided towards noxious stimuli found associations between the attentional modulation of pain intensity and activation in frontoparietal areas and the medial thalamus (Lobanov et al. 2013; Yang et al. 2018).

In summary, our findings fit well with the literature, highlighting the systems decoding’s capacity to capture and summarize brain systems involved in nociception. Some of our findings, however, stand in contrast to the traditional pain pathways described in the literature. For instance, the system components centered on right cluster 2 include visual, parietal (i.e., superior, and inferior parietal cortex), and dorsolateral prefrontal areas. Thus, in this case, the co-activations with cortical areas cannot be unambiguously associated with one of the two pathways. Notably, there is increasing evidence that partial functional overlaps exist between both the sensory-discriminative and the affective-motivational systems (Bushnell and Duncan 1989; Apkarian et al. 2011; Groh et al. 2017), which might serve as an explanation for our results. Furthermore, nociceptive processes are influenced by cognitive processes relying on widespread cortical interactions. In line with this reasoning, especially the assessment of pain intensity has been associated with both pathways, while being influenced by expectations (Atlas and Wager 2012; Lobanov et al. 2014) and attentional processes (Bantick et al. 2002; Kulkarni et al. 2005; Lobanov et al. 2013; Hauck et al. 2015). In addition, traditional research on spino-thalamico-cortical nociceptive pathways is primarily based on anatomical and/or animal studies, with known limitational factors regarding a translation to complex human cognitive functioning (Premack 2007). In conclusion, the novel aspects of our findings highlight the importance of further research with an emphasis on thalamo-cortical interactions for understanding the complexity of nociceptive processes.

#### Lateralization of thalamic clusters

The most apparent hemispheric difference was the asymmetric parcellation of the thalamus into four (left) versus three clusters (right). This might be a consequence of functional lateralization within the thalamus, which has, for instance, been documented for language: The left ventral lateral nucleus seems to be more involved in a wider set of language-related functions than its right hemispheric counterpart (Wang et al. 2021, 2022). The lateral nucleus is majorly involved in the here observed asymmetries: On the right side, the ventral lateral nucleus was grouped into a cluster with sensorimotor nuclei (cluster 2), whereas on the left side, the ventral nucleus was grouped together with higher-order nuclei (cluster 1) but not with other sensorimotor nuclei (cluster 2). Accordingly, the main functional associations for left cluster 1 were language-related (i.e., ‘linguistic’, ‘orthographic’), while the associations for right cluster 2 were mostly sensorimotor-related. While the observed thalamic asymmetry might thus be a result of functional lateralization, it remains open why other thalamic parcellations do not feature such asymmetry. One possibility would be that asymmetries are rather functional than anatomical and become only apparent when aggregating several thalamic nuclei into larger clusters. Unlike others (Yuan et al. 2017), our statistical clustering did not optimize for a symmetrical solution and might have been more sensitive to pick up functional asymmetries. Although it is easily conceivable that the asymmetric clustering of the thalamus reflects a principle of thalamo-cortical interactions in the human brain, there is also a chance that our using a corpus of neuroimaging studies has artificially inflated asymmetries: most older cognitive neuroscience experiments were conducted with right-handed participants. While there are no signs of an inherent bias in BrainMap results, there might still be a higher functional resolution in the left hemisphere, particularly when co-activations are based on tasks involving movements with the dominant hand.

### Methodological considerations

The fundamental role of the thalamus in brain functioning and behavior is unequivocal. However, to the best of our knowledge, no existing study has addressed the functional significance and the full behavioral repertoire of the thalamus and its internal structure in a human neuroimaging context. This surprising gap in the literature might stem from general methodological factors: Existing limits in spatial and functional resolution impose constrains on the ability to disambiguate signals from compact subcortical nuclei, a problem which is further amplified by the spatial blurring of signals during smoothing procedures in statistical image analysis. Quite often, the thalamus has only been studied or considered as a whole, and small nuclei such as the intralaminar ones, implicated in various cognitive functions, have remained unattainable (Saalmann 2014; Steullet 2020; Antonucci et al. 2021). Imaging protocols are also often optimized for cortical structures and might not be suitable to detect signals in subcortical areas (Keuken et al. 2018; Miletić et al. 2020). The strong focus on the cortex in cognitive neuroscience has been criticized (Johansen-Berg 2013), and recent advancements in neuroimaging were able to address some of the aforementioned shortcomings (Forstmann et al. 2017). However, there is a natural gap between recent technical developments in the field of neuroimaging and the available corpus of neuroimaging datasets in BrainMap and Neurosynth, which are a prerequisite for the systematic characterizations of a brain region’s functional profile.

We attempted to ameliorate existing constrains by deriving a novel thalamic parcellation with appropriate resolution for meta-analytic decoding that—despite its relative coarseness—aligned with known anatomical properties of the thalamus. But even with this parcellation, the established and in other contexts successful meta-analytic decoding (e.g., Clos et al. 2013; Genon et al. 2016) was not very productive in detecting cognitive or behavioral associations with the thalamus. A viable explanation would be that thalamic contributions to behavioral functions are so fundamental that the lack of specificity precludes successful decoding. But given the wide-spread connectivity between the thalamus and the neocortex and given the considerable amount of functional segregation within neocortex, it should be possible to catch a better glimpse into the thalamus’ functional repertoire by examining the thalamus and the cortex in conjunction. We have applied a novel system-level decoding approach that is based on this very idea that behavioral and cognitive processes are based on the integrative interaction of spatially segregated functional units. While this decoding approach was more successful than the traditional approach in detecting behavioral associations with the thalamus, revealed functional brain systems associated with single behavioral categories, and generated new hypotheses for future imaging research, there are still natural limitations and constrains to this approach, which we will address in the following.

Our decoding approach draws upon the Neurosynth database. It, therefore, depends fully on the database’s capability to capture the relevant cognitive neuroscience literature. Neurosynth contains studies with different experimental designs, scanner hardware, and imaging protocols, and includes older studies with small sample size, which has been discussed as a major limiting factor for reliability in functional neuroimaging (Bennett and Miller 2013; Elliott et al. 2020). While the principal strength of the meta-analytic approach is to aggregate across such detailed differences in study design, it is still not guaranteed that existing relationships are robust enough to stand against the background noise in the database. The terms in the Neurosynth database are accumulated by automatic parsing of the literature and are therefore subject to a certain ambiguity. Terms with a putative similar semantic meaning (e.g., ‘autobiographic’, ‘autobiographical memory’, ‘episodic memory’, ‘remembering’) are treated as separate, and the corpus includes many terms that defy a clear functional categorization (e.g., ‘allocation’, ‘loop’, ‘force’, ‘matrix’). Furthermore, terms such as ‘autobiographical memory’ are phenomenologically complex and researched in a variety of contexts. Thus, when defining a thalamus-centered ‘autobiographical memory’ system, without further knowledge about the research context of the term, we cannot conclude whether the corpus of studies that contain the term did focus on storage or retrieval, or the executive versus affective aspects of autobiographical memory processes, or maybe even focused on other memory processes and only contrasted them with autobiographical ones.

Furthermore, our decoding approach rests on the validity of the thalamic parcellation. While our parcellation’s correspondence with known cytoarchitecture and its optimization for task-evoked co-activations provide ample arguments for its validity in the context of our present work, it still needs to be pointed out that it relies on the statistical clustering of information from one imaging modality. Given that the accuracy of brain parcellations benefits from the inclusion of multi-modal imaging (Glasser et al. 2016), there is certainly room for adjustments when parcellating the human thalamus in future studies.

Given these limitations, we urge to see the present work as a major but far-from-final step towards objectively characterizing the behavioral repertoire of the thalamus from a systems perspective, based on a large corpus of neuroimaging studies. As such, besides providing new answers, it generated several hypotheses*.* For instance, the observed functional overlap between thalamic clusters regarding the thalamo-centered part of the autobiographical memory and the nociceptive systems, raises the question of whether this indicates separate systems that might participate in different aspects of autobiographical memory, or whether this is a consequence of not fully separable signals from the different clusters. We, therefore, encourage to take advantage of recent improvements in resolution (e.g., ultra-high-field 7 T MRI) and imaging protocols (e.g., multi-echo EPI protocols), when examining thalamic subdivisions in future studies. Finally, the here reported structure–function associations do not tell us anything about the underlying computational mechanisms of thalamic involvement in cognitive functioning. Further experimental and computational work is needed to explain the role of thalamo-cortical co-activation in a given behavioral domain.

### Summary

Through a novel data-driven parcellation of the thalamus and a novel systems-decoding strategy, we were able to derive new insights into the behavioral relevance of thalamic subunits in interactions with cortical areas. Our results are in line with known models of thalamic function, emphasize the role of the thalamus in cortical functioning, and point to as-yet unnoticed aspects of thalamo-cortical functioning, for instance, the thalamo-parietal connections in autobiographical memory. We propose the systems-level decoding as a hypothesis-generating approach for functional connectivity studies and a complementary approach that is easy enough to implement when interpreting existing functional connectivity studies.

### Supplementary Information

Below is the link to the electronic supplementary material.

Supplementary file1 (PDF 6452 KB)

## Data Availability

The datasets generated during the current study will be publicly available in the ANIMA database (https://anima.fz-juelich.de/, Reid et al. 2016) upon publication of the manuscript and will be otherwise available from the corresponding author on reasonable request.
