# Supplementary material for: Characterizing functional modules in the human thalamus: coactivation-based parcellation and systems-level functional decoding
Source: Brain Struct Funct. 2022 Dec 22;228(8):1811–34. doi: 10.1007/s00429-022-02603-w (PMC10516793; doi:10.1007/s00429-022-02603-w)
Supplement: Supplementary file 1 — Supplementary file1 (PDF 6452 KB) [file 429_2022_2603_MOESM1_ESM.pdf]

Title: Characterizing functional modules in the human thalamus: Coactivation-based parcellation and systems-level functional decoding

Journal: Special Issue: Update Brain Parcellations and their Relationship to Function, Brain Structure and Function

Author(s): Ole J. Boeken, Edna C. Cieslik, Robert Langner, Sebastian Markett

Corresponding author:

Ole Jonas Boeken

Humboldt-University Berlin, Department of Molecular Psychology, Berlin, Germany

E-Mail: olejonas.boeken@gmail.com

Supplementary Information

In this document, we present supplementary information on the parcellation approach, the brain atlases used, detailed information on the decoding results, and an alternative systems-level decoding constrained by coactivations.

S1. Visualization of optimal filter range

S2. Supplementary information on optimal cluster selection

S3. Visualization of cluster selection criteria

S4. Overview of brain regions in the cortical parcellation

S5. Illustration of cortical parcellation

S6. Decoding results: Neurosynth topics for thalamic clusters

S7. Supplementary analysis: Coactivation-constrained systems-level decoding

S8. Illustration of cortical topology of coactivations

S9. Supplementary Results: Systems

S10: Comparison of main and supplemental systems-level decoding

S11 + S12: Differences between main and supplemental systems-level decoding

S13: Comparison of the MACM-CBP parcellation with the Morel atlas

S14: Illustration of Morel atlas and major thalamic nuclei

S15: Supplementary Results: Top terms in the standard decoding

S16: Supplementary Results: Systems-level Decoding

S17: Results from supplementary systems-level decoding

Supplementary Figure S1, Selection of optimal filter range: Illustration of the proportion of deviants (normalized within each cluster solution k) for the (A) left and (B) right thalamus. Vertical lines indicate the selected, most stable, range of filter sizes comprising the lowest number of deviants (i.e. voxels assigned differently as compared to the solution from the majority of filters). For left thalamus optimal filter size ranged from 104-156 experiments and for the right thalamus optimal filter size ranged from 106-154 experiments.

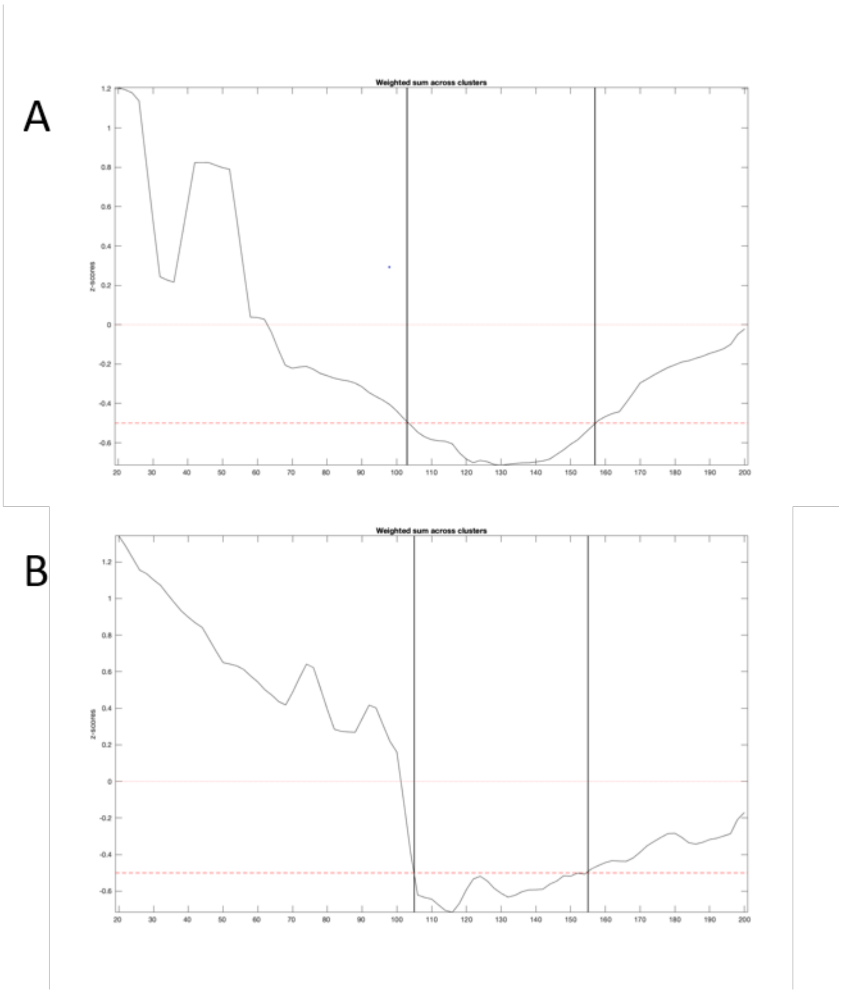

Supplementary Information S2: Selection of optimal cluster solution

As in previous studies (e.g. (Clos et al. 2013; Genon et al. 2016; Plachti et al. 2019) the optimal most stable cluster solution for the k-means clustering was selected based on different criteria including topological, information-theoretic and cluster separation characteristics.

Three topological criteria were taken into account, i) *the detection of the percentage of misclassified voxels (i.e., deviants)*, ii) *voxels not related to the dominant parent cluster (i.e., related to the hierarchy index*) and iii) *the number of consistent voxels per cluster*. The *detection of the percentage of misclassified voxels* addresses the across-filter stability, i.e. the average percentage of voxels for each

filter-size that are assigned differentially (i.e. assigned to a different cluster compared to the most frequent assignment of these voxels across all filter sizes). Optimal cluster solutions are considered those k parcellations where the percentage of deviants is not significantly increased (tested with a two-sample t-test) compared to the previous (k-1) cluster solution, in particular if the subsequent (k+1) solution leads to a significantly higher percentage of deviants.

The criteria considering the percentage of *voxels not related to the dominant parent cluster* compared to the previous cluster solution is related to the hierarchy-index (Kahnt et al. 2012). Here, voxels that are assigned to e.g. cluster 3 in the k = 3 solution deriving from a subset of voxels previously assigned to cluster 2 (in the k = 2 solution) would be excluded if the majority of cluster 3 voxels actually stemmed from cluster 1 (in the k = 2 solution). Optimal cluster solutions are considered those where the percentage of these hierarchically inconsistent and hence excluded voxels is below the median across all possible cluster solutions, where the respective clustering step represents a local minimum, or where the following cluster solution represents a maximum in the percentage of hierarchically inconsistent voxels.

The third topological criteria, *the number of consistent voxels per cluster*, represents the size of the individual cluster after removing hierarchically inconsistent voxels. For each cluster solution the proportion of the minimum cluster size is compared to the mean cluster size and optimal cluster solutions are considered those where the size of the minimum cluster size is more than half of the average cluster size.

As an information-theoretic criteria *variation of information (VI)* was used. This criterion quantifies the amount of information gained and lost in changing from one clustering solution to another (Meilă 2007). Hereby, for each cluster solution, VI between all combination of the previously selected *x* filter sizes (e.g. 27 filter sizes (104,106, 108…156) for left thalamus) was computed. A two-sample t-test was used to test for significant differences in VI between a given and the subsequent clustering step. Optimal clustering steps were considered those that did not show an increase in *VI between filter sizes* compared to the previous k-1 solution or that showed a significant increase in VI for the k+1 solution. Further, *variation of information across cluster* was determined, with good cluster solutions being those that showed a significant increase in VI from the current to the k+1 clustering step or a significant decrease in VI from the previous to the current clustering step.

Finally, cluster separation criteria were also included to determine the most stable clustering solution. Here, *information on cluster separation* was computed as the intercluster to intracluster distance ratio

(Chang et al. 2013) for the filter size selected. This criterion describes the ratio between the average distance of a voxel to its cluster centre and the average distance between the cluster centres. Here, good solutions are considered those where this ratio is increased compared to the previous k-1 solution. Additionally, the first derivative of this criterion is used for evaluating the change in this ratio compared

to the previous k-1 solution, with good solutions showing no significant larger increase in intercluster to intracluster distance when moving to the k+1 solution.

In addition, as done in previous CBP studies (Kelly et al. 2010; Genon et al. 2016) the *silhouette value* averaged across voxels for each filter size in the previously selected filter range was computed. This criterion describes how similar a voxel is compared to voxels within its own cluster versus how similar it is to voxels within other clusters regarding its co-activation profile, with values ranges from -1 to +1. A two-sample t-test is used to test for significant differences in the silhouette value between the present cluster solution compared to the previous one. Stable cluster solutions are considered those that show a significantly higher silhouette value compared to the k-1 solution (primary criterion), or those solutions that do not show a significantly lower silhouette value compared to the k-1 solution (secondary criterion).

Supplementary Figure S3: Selection of optimal cluster solution was performed based on topological, information-theoretic as well as cluster separation criteria. Taking into consideration all criteria, for the left thalamus a parcellation into four cluster is the one with the highest share of support.

Topological criteria

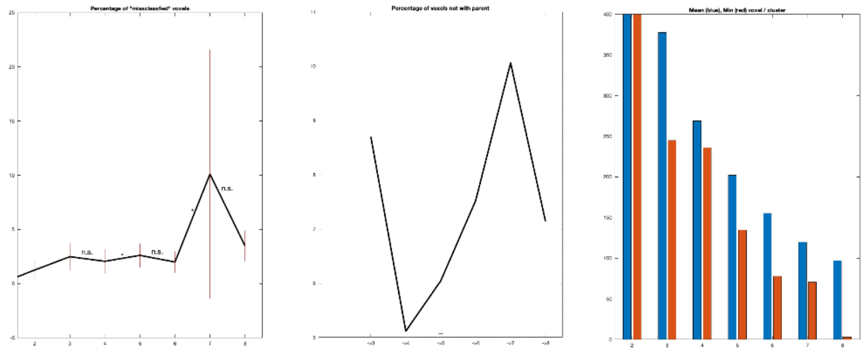

Information-theoretic criteria

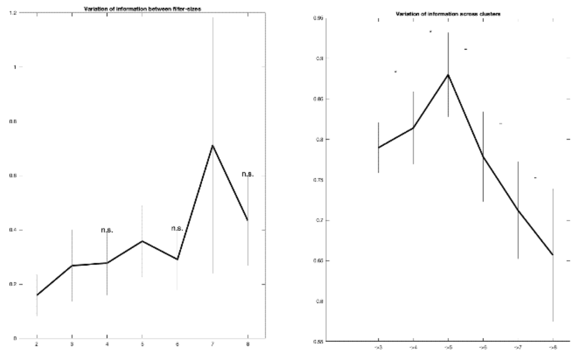

Cluster separation criteria:

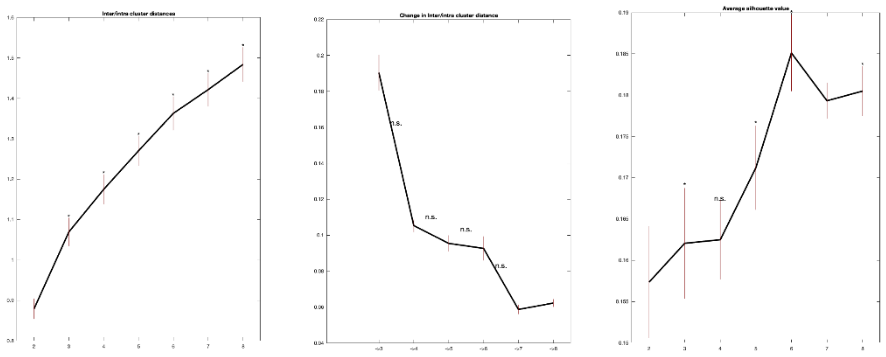

Right thalamus: For the right thalamus a parcellation into three cluster is identified as an optimal clustering solution when taking into account all criteria.

Topological criteria

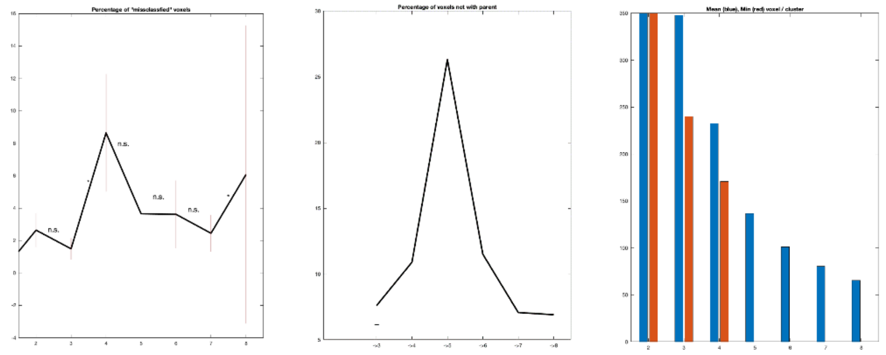

Information-theoretic criteria

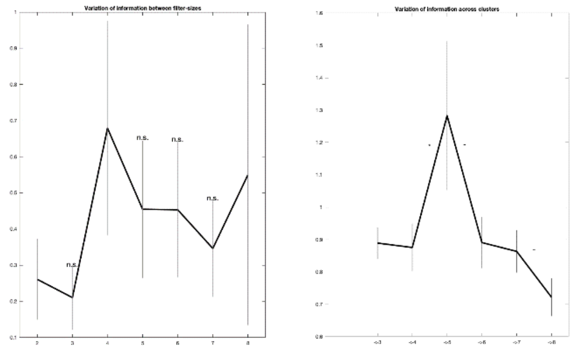

Cluster separation criteria

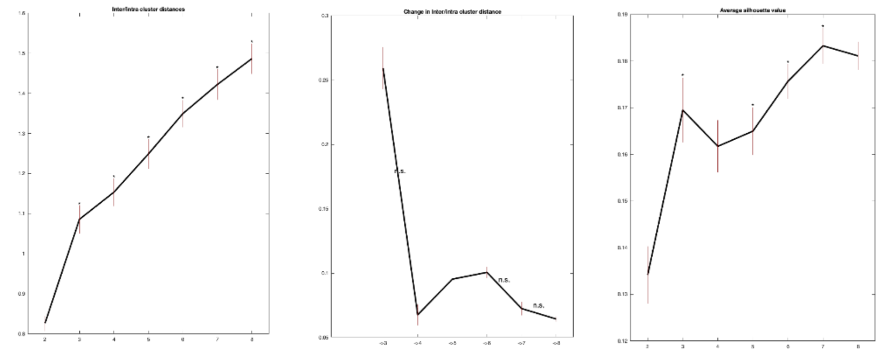

References:

Chang LJ, Yarkoni T, Khaw MW, Sanfey AG (2013) Decoding the Role of the Insula in Human Cognition: Functional Parcellation and Large-Scale Reverse Inference. Cereb Cortex 23:739–749. https://doi.org/10.1093/cercor/bhs065

Clos M, Amunts K, Laird AR, et al (2013) Tackling the multifunctional nature of Broca’s region meta-analytically: Co-activation-based parcellation of area 44. NeuroImage 83:174–188. https://doi.org/10.1016/j.neuroimage.2013.06.041

Genon S, Li H, Fan L, et al (2016) The Right Dorsal Premotor Mosaic: Organization, Functions, and Connectivity. Cereb Cortex bhw065. https://doi.org/10.1093/cercor/bhw065

Kahnt T, Chang LJ, Park SQ, et al (2012) Connectivity-Based Parcellation of the Human Orbitofrontal Cortex. J Neurosci 32:6240–6250. https://doi.org/10.1523/JNEUROSCI.0257-12.2012

Kelly C, Uddin LQ, Shehzad Z, et al (2010) Broca’s region: linking human brain functional connectivity data and non-human primate tracing anatomy studies: Functional connectivity of Broca’s region. Eur J Neurosci 32:383–398. https://doi.org/10.1111/j.1460-9568.2010.07279.x

Meilă M (2007) Comparing clusterings—an information based distance. J Multivar Anal 98:873–895. https://doi.org/10.1016/j.jmva.2006.11.013

Plachti A, Eickhoff SB, Hoffstaedter F, et al (2019) Multimodal Parcellations and Extensive Behavioral Profiling Tackling the Hippocampus Gradient. Cereb Cortex 29:4595–4612. https://doi.org/10.1093/cercor/bhy336

Supplementary Table S4: Complete list of all HCP-MMP (Glasser et al. 2016) regions, their cortical ID’s and region names

**regionID**	**regionName**	**regionLongName**
1	**V1_L**	Primary_Visual_Cortex_L
2	**MST_L**	Medial_Superior_Temporal_Area_L
3	**V6_L**	Sixth_Visual_Area_L
4	**V2_L**	Second_Visual_Area_L
5	**V3_L**	Third_Visual_Area_L
6	**V4_L**	Fourth_Visual_Area_L

7	**V8_L**	Eighth_Visual_Area_L
8	**4_L**	Primary_Motor_Cortex_L
9	**3b_L**	Primary_Sensory_Cortex_L
10	**FEF_L**	Frontal_Eye_Fields_L
11	**PEF_L**	Premotor_Eye_Field_L
12	**55b_L**	Area_55b_L
13	**V3A_L**	Area_V3A_L
14	**RSC_L**	RetroSplenial_Complex_L
15	**POS2_L**	Parieto-Occipital_Sulcus_Area_2_L
16	**V7_L**	Seventh_Visual_Area_L
17	**IPS1_L**	IntraParietal_Sulcus_Area_1_L
18	**FFC_L**	Fusiform_Face_Complex_L
19	**V3B_L**	Area_V3B_L
20	**LO1_L**	Area_Lateral_Occipital_1_L
21	**LO2_L**	Area_Lateral_Occipital_2_L
22	**PIT_L**	Posterior_InferoTemporal_complex_L
23	**MT_L**	Middle_Temporal_Area_L
24	**A1_L**	Primary_Auditory_Cortex_L
25	**PSL_L**	PeriSylvian_Language_Area_L
26	**SFL_L**	Superior_Frontal_Language_Area_L
27	**PCV_L**	PreCuneus_Visual_Area_L
28	**STV_L**	Superior_Temporal_Visual_Area_L
29	**7Pm_L**	Medial_Area_7P_L
30	**7m_L**	Area_7m_L
31	**POS1_L**	Parieto-Occipital_Sulcus_Area_1_L
32	**23d_L**	Area_23d_L
33	**v23ab_L**	Area_ventral_23_a+b_L
34	**d23ab_L**	Area_dorsal_23_a+b_L
35	**31pv_L**	Area_31p_ventral_L
36	**5m_L**	Area_5m_L
37	**5mv_L**	Area_5m_ventral_L
38	**23c_L**	Area_23c_L
39	**5L_L**	Area_5L_L
40	**24dd_L**	Dorsal_Area_24d_L
41	**24dv_L**	Ventral_Area_24d_L
42	**7AL_L**	Lateral_Area_7A_L
43	**SCEF_L**	Supplementary_and_Cingulate_Eye_Field_L
44	**6ma_L**	Area_6m_anterior_L
45	**7Am_L**	Medial_Area_7A_L
46	**7Pl_L**	Lateral_Area_7P_L
47	**7PC_L**	Area_7PC_L
48	**LIPv_L**	Area_Lateral_IntraParietal_ventral_L
49	**VIP_L**	Ventral_IntraParietal_Complex_L
50	**MIP_L**	Medial_IntraParietal_Area_L
51	**1_L**	Area_1_L

52	**2_L**	Area_2_L
53	**3a_L**	Area_3a_L
54	**6d_L**	Dorsal_area_6_L
55	**6mp_L**	Area_6mp_L
56	**6v_L**	Ventral_Area_6_L
57	**p24pr_L**	Area_Posterior_24_prime_L
58	**33pr_L**	Area_33_prime_L
59	**a24pr_L**	Anterior_24_prime_L
60	**p32pr_L**	Area_p32_prime_L
61	**a24_L**	Area_a24_L
62	**d32_L**	Area_dorsal_32_L
63	**8BM_L**	Area_8BM_L
64	**p32_L**	Area_p32_L
65	**10r_L**	Area_10r_L
66	**47m_L**	Area_47m_L
67	**8Av_L**	Area_8Av_L
68	**8Ad_L**	Area_8Ad_L
69	**9m_L**	Area_9_Middle_L
70	**8BL_L**	Area_8B_Lateral_L
71	**9p_L**	Area_9_Posterior_L
72	**10d_L**	Area_10d_L
73	**8C_L**	Area_8C_L
74	**44_L**	Area_44_L
75	**45_L**	Area_45_L
76	**47l_L**	Area_47l_(47_lateral)_L
77	**a47r_L**	Area_anterior_47r_L
78	**6r_L**	Rostral_Area_6_L
79	**IFJa_L**	Area_IFJa_L
80	**IFJp_L**	Area_IFJp_L
81	**IFSp_L**	Area_IFSp_L
82	**IFSa_L**	Area_IFSa_L
83	**p9-46v_L**	Area_posterior_9-46v_L
84	**46_L**	Area_46_L
85	**a9-46v_L**	Area_anterior_9-46v_L
86	**9-46d_L**	Area_9-46d_L
87	**9a_L**	Area_9_anterior_L
88	**10v_L**	Area_10v_L
89	**a10p_L**	Area_anterior_10p_L
90	**10pp_L**	Polar_10p_L
91	**11l_L**	Area_11l_L
92	**13l_L**	Area_13l_L
93	**OFC_L**	Orbital_Frontal_Complex_L
94	**47s_L**	Area_47s_L
95	**LIPd_L**	Area_Lateral_IntraParietal_dorsal_L
96	**6a_L**	Area_6_anterior_L

97	**i6-8_L**	Inferior_6-8_Transitional_Area_L
98	**s6-8_L**	Superior_6-8_Transitional_Area_L
99	**43_L**	Area_43_L
100	**OP4_L**	Area_OP4-PV_L
101	**OP1_L**	Area_OP1-SII_L
102	**OP2-3_L**	Area_OP2-3-VS_L
103	**52_L**	Area_52_L
104	**RI_L**	RetroInsular_Cortex_L
105	**PFcm_L**	Area_PFcm_L
106	**PoI2_L**	Posterior_Insular_Area_2_L
107	**TA2_L**	Area_TA2_L
108	**FOP4_L**	Frontal_Opercular_Area_4_L
109	**MI_L**	Middle_Insular_Area_L
110	**Pir_L**	Pirform_Cortex_L
111	**AVI_L**	Anterior_Ventral_Insular_Area_L
112	**AAIC_L**	Anterior_Agranular_Insula_Complex_L
113	**FOP1_L**	Frontal_Opercular_Area_1_L
114	**FOP3_L**	Frontal_Opercular_Area_3_L
115	**FOP2_L**	Frontal_Opercular_Area_2_L
116	**PFt_L**	Area_PFt_L
117	**AIP_L**	Anterior_IntraParietal_Area_L
118	**EC_L**	Entorhinal_Cortex_L
119	**PreS_L**	PreSubiculum_L
120	**H_L**	Hippocampus_L
121	**ProS_L**	ProStriate_Area_L
122	**PeEc_L**	Perirhinal_Ectorhinal_Cortex_L
123	**STGa_L**	Area_STGa_L
124	**PBelt_L**	ParaBelt_Complex_L
125	**A5_L**	Auditory_5_Complex_L
126	**PHA1_L**	ParaHippocampal_Area_1_L
127	**PHA3_L**	ParaHippocampal_Area_3_L
128	**STSda_L**	Area_STSd_anterior_L
129	**STSdp_L**	Area_STSd_posterior_L
130	**STSvp_L**	Area_STSv_posterior_L
131	**TGd_L**	Area_TG_dorsal_L
132	**TE1a_L**	Area_TE1_anterior_L
133	**TE1p_L**	Area_TE1_posterior_L
134	**TE2a_L**	Area_TE2_anterior_L
135	**TF_L**	Area_TF_L
136	**TE2p_L**	Area_TE2_posterior_L
137	**PHT_L**	Area_PHT_L
138	**PH_L**	Area_PH_L
139	**TPOJ1_L**	Area_TemporoParietoOccipital_Junction_1_L
140	**TPOJ2_L**	Area_TemporoParietoOccipital_Junction_2_L
141	**TPOJ3_L**	Area_TemporoParietoOccipital_Junction_3_L

142	**DVT_L**	Dorsal_Transitional_Visual_Area_L
143	**PGp_L**	Area_PGp_L
144	**IP2_L**	Area_IntraParietal_2_L
145	**IP1_L**	Area_IntraParietal_1_L
146	**IP0_L**	Area_IntraParietal_0_L
147	**PFop_L**	Area_PF_Opercular_L
148	**PF_L**	Area_PF_Complex_L
149	**PFm_L**	Area_PFm_Complex_L
150	**PGi_L**	Area_PGi_L
151	**PGs_L**	Area_PGs_L
152	**V6A_L**	Area_V6A_L
153	**VMV1_L**	VentroMedial_Visual_Area_1_L
154	**VMV3_L**	VentroMedial_Visual_Area_3_L
155	**PHA2_L**	ParaHippocampal_Area_2_L
156	**V4t_L**	Area_V4t_L
157	**FST_L**	Area_FST_L
158	**V3CD_L**	Area_V3CD_L
159	**LO3_L**	Area_Lateral_Occipital_3_L
160	**VMV2_L**	VentroMedial_Visual_Area_2_L
161	**31pd_L**	Area_31pd_L
162	**31a_L**	Area_31a_L
163	**VVC_L**	Ventral_Visual_Complex_L
164	**25_L**	Area_25_L
165	**s32_L**	Area_s32_L
166	**pOFC_L**	Posterior_OFC_Complex_L
167	**PoI1_L**	Area_Posterior_Insular_1_L
168	**Ig_L**	Insular_Granular_Complex_L
169	**FOP5_L**	Area_Frontal_Opercular_5_L
170	**p10p_L**	Area_posterior_10p_L
171	**p47r_L**	Area_posterior_47r_L
172	**TGv_L**	Area_TG_Ventral_L
173	**MBelt_L**	Medial_Belt_Complex_L
174	**LBelt_L**	Lateral_Belt_Complex_L
175	**A4_L**	Auditory_4_Complex_L
176	**STSva_L**	Area_STSv_anterior_L
177	**TE1m_L**	Area_TE1_Middle_L
178	**PI_L**	Para-Insular_Area_L
179	**a32pr_L**	Area_anterior_32_prime_L
180	**p24_L**	Area_posterior_24_L
201	**V1_R**	Primary_Visual_Cortex_R
202	**MST_R**	Medial_Superior_Temporal_Area_R
203	**V6_R**	Sixth_Visual_Area_R
204	**V2_R**	Second_Visual_Area_R
205	**V3_R**	Third_Visual_Area_R
206	**V4_R**	Fourth_Visual_Area_R

207	**V8_R**	Eighth_Visual_Area_R
208	**4_R**	Primary_Motor_Cortex_R
209	**3b_R**	Primary_Sensory_Cortex_R
210	**FEF_R**	Frontal_Eye_Fields_R
211	**PEF_R**	Premotor_Eye_Field_R
212	**55b_R**	Area_55b_R
213	**V3A_R**	Area_V3A_R
214	**RSC_R**	RetroSplenial_Complex_R
215	**POS2_R**	Parieto-Occipital_Sulcus_Area_2_R
216	**V7_R**	Seventh_Visual_Area_R
217	**IPS1_R**	IntraParietal_Sulcus_Area_1_R
218	**FFC_R**	Fusiform_Face_Complex_R
219	**V3B_R**	Area_V3B_R
220	**LO1_R**	Area_Lateral_Occipital_1_R
221	**LO2_R**	Area_Lateral_Occipital_2_R
222	**PIT_R**	Posterior_InferoTemporal_complex_R
223	**MT_R**	Middle_Temporal_Area_R
224	**A1_R**	Primary_Auditory_Cortex_R
225	**PSL_R**	PeriSylvian_Language_Area_R
226	**SFL_R**	Superior_Frontal_Language_Area_R
227	**PCV_R**	PreCuneus_Visual_Area_R
228	**STV_R**	Superior_Temporal_Visual_Area_R
229	**7Pm_R**	Medial_Area_7P_R
230	**7m_R**	Area_7m_R
231	**POS1_R**	Parieto-Occipital_Sulcus_Area_1_R
232	**23d_R**	Area_23d_R
233	**v23ab_R**	Area_ventral_23_a+b_R
234	**d23ab_R**	Area_dorsal_23_a+b_R
235	**31pv_R**	Area_31p_ventral_R
236	**5m_R**	Area_5m_R
237	**5mv_R**	Area_5m_ventral_R
238	**23c_R**	Area_23c_R
239	**5L_R**	Area_5L_R
240	**24dd_R**	Dorsal_Area_24d_R
241	**24dv_R**	Ventral_Area_24d_R
242	**7AL_R**	Lateral_Area_7A_R
243	**SCEF_R**	Supplementary_and_Cingulate_Eye_Field_R
244	**6ma_R**	Area_6m_anterior_R
245	**7Am_R**	Medial_Area_7A_R
246	**7Pl_R**	Lateral_Area_7P_R
247	**7PC_R**	Area_7PC_R
248	**LIPv_R**	Area_Lateral_IntraParietal_ventral_R
249	**VIP_R**	Ventral_IntraParietal_Complex_R
250	**MIP_R**	Medial_IntraParietal_Area_R
251	**1_R**	Area_1_R

252	**2_R**	Area_2_R
253	**3a_R**	Area_3a_R
254	**6d_R**	Dorsal_area_6_R
255	**6mp_R**	Area_6mp_R
256	**6v_R**	Ventral_Area_6_R
257	**p24pr_R**	Area_Posterior_24_prime_R
258	**33pr_R**	Area_33_prime_R
259	**a24pr_R**	Anterior_24_prime_R
260	**p32pr_R**	Area_p32_prime_R
261	**a24_R**	Area_a24_R
262	**d32_R**	Area_dorsal_32_R
263	**8BM_R**	Area_8BM_R
264	**p32_R**	Area_p32_R
265	**10r_R**	Area_10r_R
266	**47m_R**	Area_47m_R
267	**8Av_R**	Area_8Av_R
268	**8Ad_R**	Area_8Ad_R
269	**9m_R**	Area_9_Middle_R
270	**8BL_R**	Area_8B_Lateral_R
271	**9p_R**	Area_9_Posterior_R
272	**10d_R**	Area_10d_R
273	**8C_R**	Area_8C_R
274	**44_R**	Area_44_R
275	**45_R**	Area_45_R
276	**47l_R**	Area_47l_(47_lateral)_R
277	**a47r_R**	Area_anterior_47r_R
278	**6r_R**	Rostral_Area_6_R
279	**IFJa_R**	Area_IFJa_R
280	**IFJp_R**	Area_IFJp_R
281	**IFSp_R**	Area_IFSp_R
282	**IFSa_R**	Area_IFSa_R
283	**p9-46v_R**	Area_posterior_9-46v_R
284	**46_R**	Area_46_R
285	**a9-46v_R**	Area_anterior_9-46v_R
286	**9-46d_R**	Area_9-46d_R
287	**9a_R**	Area_9_anterior_R
288	**10v_R**	Area_10v_R
289	**a10p_R**	Area_anterior_10p_R
290	**10pp_R**	Polar_10p_R
291	**11l_R**	Area_11l_R
292	**13l_R**	Area_13l_R
293	**OFC_R**	Orbital_Frontal_Complex_R
294	**47s_R**	Area_47s_R
295	**LIPd_R**	Area_Lateral_IntraParietal_dorsal_R
296	**6a_R**	Area_6_anterior_R

297	**i6-8_R**	Inferior_6-8_Transitional_Area_R
298	**s6-8_R**	Superior_6-8_Transitional_Area_R
299	**43_R**	Area_43_R
300	**OP4_R**	Area_OP4-PV_R
301	**OP1_R**	Area_OP1-SII_R
302	**OP2-3_R**	Area_OP2-3-VS_R
303	**52_R**	Area_52_R
304	**RI_R**	RetroInsular_Cortex_R
305	**PFcm_R**	Area_PFcm_R
306	**PoI2_R**	Posterior_Insular_Area_2_R
307	**TA2_R**	Area_TA2_R
308	**FOP4_R**	Frontal_Opercular_Area_4_R
309	**MI_R**	Middle_Insular_Area_R
310	**Pir_R**	Pirform_Cortex_R
311	**AVI_R**	Anterior_Ventral_Insular_Area_R
312	**AAIC_R**	Anterior_Agranular_Insula_Complex_R
313	**FOP1_R**	Frontal_Opercular_Area_1_R
314	**FOP3_R**	Frontal_Opercular_Area_3_R
315	**FOP2_R**	Frontal_Opercular_Area_2_R
316	**PFt_R**	Area_PFt_R
317	**AIP_R**	Anterior_IntraParietal_Area_R
318	**EC_R**	Entorhinal_Cortex_R
319	**PreS_R**	PreSubiculum_R
320	**H_R**	Hippocampus_R
321	**ProS_R**	ProStriate_Area_R
322	**PeEc_R**	Perirhinal_Ectorhinal_Cortex_R
323	**STGa_R**	Area_STGa_R
324	**PBelt_R**	ParaBelt_Complex_R
325	**A5_R**	Auditory_5_Complex_R
326	**PHA1_R**	ParaHippocampal_Area_1_R
327	**PHA3_R**	ParaHippocampal_Area_3_R
328	**STSda_R**	Area_STSd_anterior_R
329	**STSdp_R**	Area_STSd_posterior_R
330	**STSvp_R**	Area_STSv_posterior_R
331	**TGd_R**	Area_TG_dorsal_R
332	**TE1a_R**	Area_TE1_anterior_R
333	**TE1p_R**	Area_TE1_posterior_R
334	**TE2a_R**	Area_TE2_anterior_R
335	**TF_R**	Area_TF_R
336	**TE2p_R**	Area_TE2_posterior_R
337	**PHT_R**	Area_PHT_R
338	**PH_R**	Area_PH_R
339	**TPOJ1_R**	Area_TemporoParietoOccipital_Junction_1_R
340	**TPOJ2_R**	Area_TemporoParietoOccipital_Junction_2_R
341	**TPOJ3_R**	Area_TemporoParietoOccipital_Junction_3_R

342	**DVT_R**	Dorsal_Transitional_Visual_Area_R
343	**PGp_R**	Area_PGp_R
344	**IP2_R**	Area_IntraParietal_2_R
345	**IP1_R**	Area_IntraParietal_1_R
346	**IP0_R**	Area_IntraParietal_0_R
347	**PFop_R**	Area_PF_Opercular_R
348	**PF_R**	Area_PF_Complex_R
349	**PFm_R**	Area_PFm_Complex_R
350	**PGi_R**	Area_PGi_R
351	**PGs_R**	Area_PGs_R
352	**V6A_R**	Area_V6A_R
353	**VMV1_R**	VentroMedial_Visual_Area_1_R
354	**VMV3_R**	VentroMedial_Visual_Area_3_R
355	**PHA2_R**	ParaHippocampal_Area_2_R
356	**V4t_R**	Area_V4t_R
357	**FST_R**	Area_FST_R
358	**V3CD_R**	Area_V3CD_R
359	**LO3_R**	Area_Lateral_Occipital_3_R
360	**VMV2_R**	VentroMedial_Visual_Area_2_R
361	**31pd_R**	Area_31pd_R
362	**31a_R**	Area_31a_R
363	**VVC_R**	Ventral_Visual_Complex_R
364	**25_R**	Area_25_R
365	**s32_R**	Area_s32_R
366	**pOFC_R**	posterior_OFC_Complex_R
367	**PoI1_R**	Area_Posterior_Insular_1_R
368	**Ig_R**	Insular_Granular_Complex_R
369	**FOP5_R**	Area_Frontal_Opercular_5_R
370	**p10p_R**	Area_posterior_10p_R
371	**p47r_R**	Area_posterior_47r_R
372	**TGv_R**	Area_TG_Ventral_R
373	**MBelt_R**	Medial_Belt_Complex_R
374	**LBelt_R**	Lateral_Belt_Complex_R
375	**A4_R**	Auditory_4_Complex_R
376	**STSva_R**	Area_STSv_anterior_R
377	**TE1m_R**	Area_TE1_Middle_R
378	**PI_R**	Para-Insular_Area_R
379	**a32pr_R**	Area_anterior_32_prime_R
380	**p24_R**	Area_posterior_24_R

References:

Glasser MF, Coalson TS, Robinson EC, et al (2016) A multi-modal parcellation of human cerebral cortex. Nature 536:171–178. https://doi.org/10.1038/nature18933

Supplementary Figure S5: Surface renderings of the 360 cortical regions of the HCP-MMP atlas used in the present study. At the top we depict the label annotations, overlaid with the outlines of the HCP-MMP atlas parcellation. At the bottom, we display the color graded 360 cortical regions. For a detailed list of the cortical regions, the reader is referred to S4.

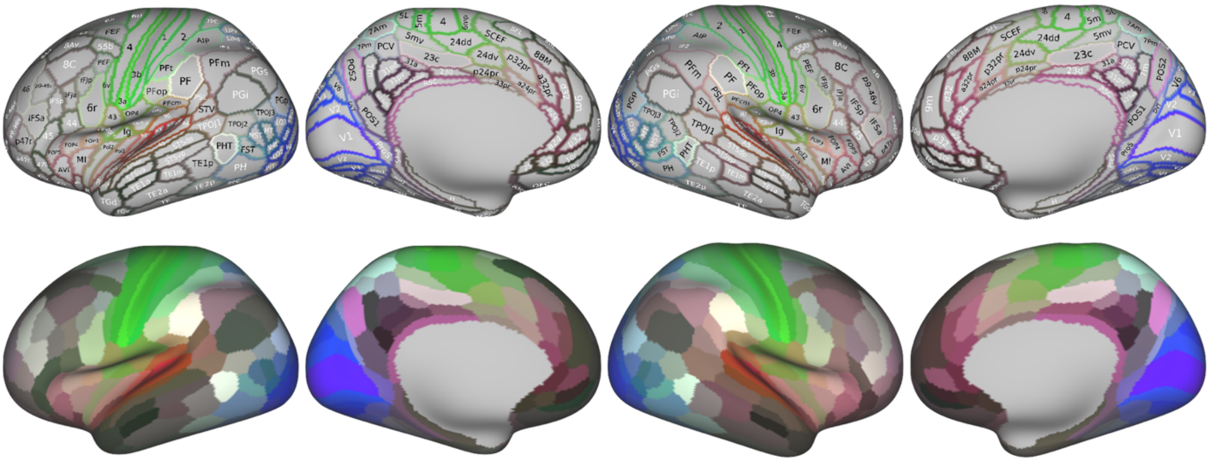

References:

Glasser MF, Coalson TS, Robinson EC, et al (2016) A multi-modal parcellation of human cerebral cortex. Nature 536:171–178. https://doi.org/10.1038/nature18933

Supplementary Figure S6: Heatmaps of the systems-level terms, aggregated into larger topics for each thalamic cluster. Here, we display all systems-level decoding terms denoting psychological constructs grouped into a given topic. The visualization is hierarchically ordered by the number of term appearances across the thalamic clusters. The terms were grouped into the most recent version of 50 topics as provided by Neurosynth, by testing whether or not a given term is listed in a particular topic. Of note, the 50 topics terms are presented as single terms (e.g., 'finger'), whereas the Neurosynth corpus of terms can consist of compound terms (e.g., 'finger tapping'). We therefore split the compound terms into two components ('i.e., 'finger', 'tapping') and grouped them only into a particular topic together if both of the sub-components were listed in the topic.

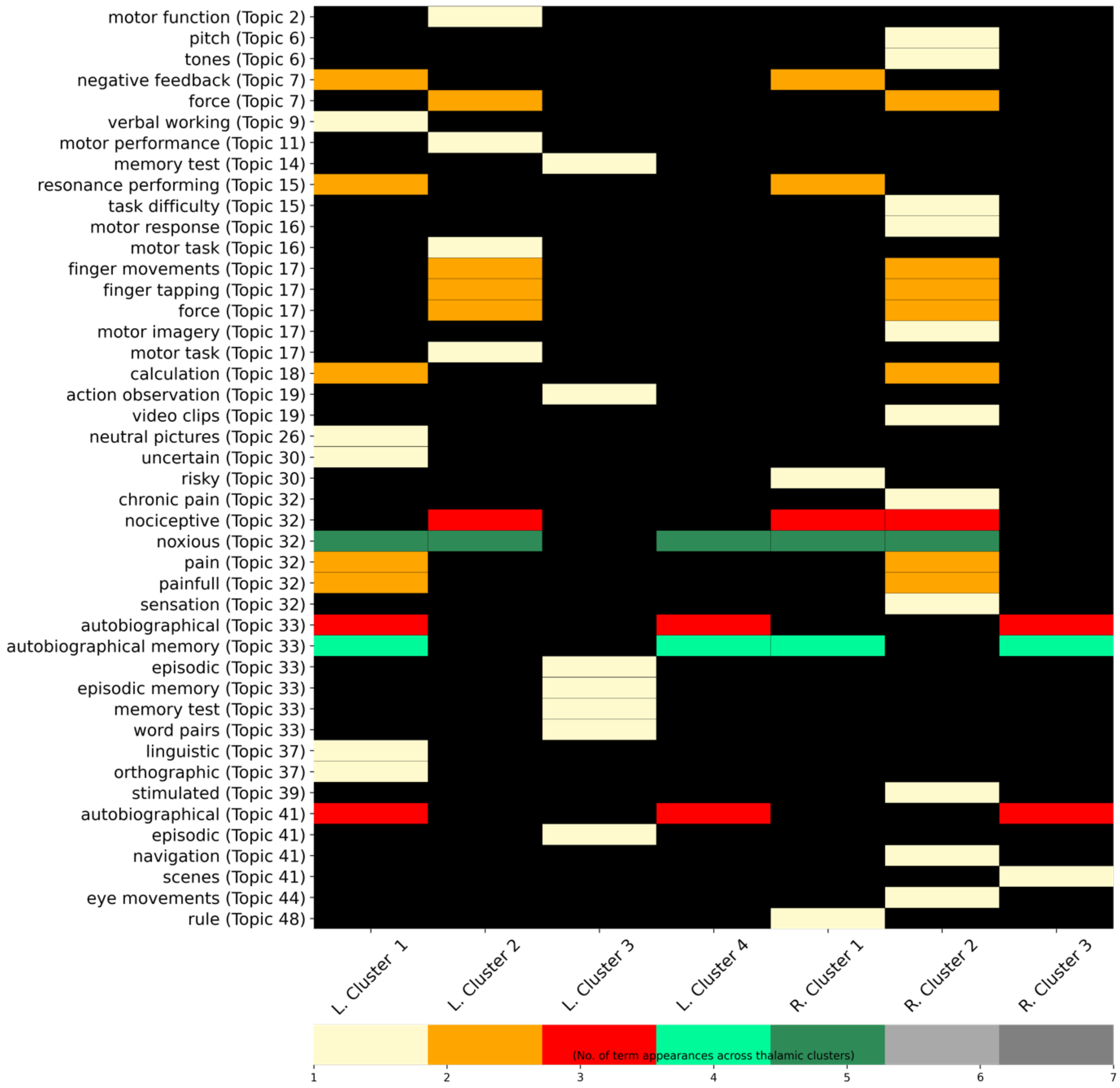

Supplementary Information S7: Constraining the cortical regions associated with a given cluster to detect functional brain systems via the systems-level decoding was done with resting state functional connectivity. However, the MACM analysis also provided task-based coactivation maps for all thalamic clusters. To validate the novel systems-level decoding, we performed a supplementary validation analysis. This time we used task-constraint coactivation maps from the MACM-analysis to prioritize candidate cortical regions for the systems-level decoding. Thalamo-cortical pairs were taken from FWE-corrected task-constraint coactivation maps (thresholded at $p < 0.05$, cluster level FWE-corrected, cluster-forming threshold: $p < 0.001$ on the voxel level, as done in previous studies (Genon et al., 2016)) that were produced by an ALE meta-analysis, performed across all BrainMap experiments with at least one activation focus featured within a given particular cluster (see methods section). For the supplementary systems-level decoding we merged the mask of a cluster with the foci from the HCP-MMP regions that showed an overlap with a given coactivation map (as opposed to significant resting-

state functional connectivity in the main analysis). For each individual cluster, a HCP-MMP region was only selected, if that region surpassed a threshold: The threshold was determined by the knee of a curve after rank-ordering cortical regions according to their spatial overlap with the coactivation map. All resulting thalamo-cortical pairs were then entered to the database query and the whole systems-level decoding procedure repeated for all clusters in conjunction with their task-constrained coactivated regions.

The main difference between the main and supplementary systems-level decoding was a different pre-selection of cortical regions. The main analysis was performed on 226 cortical regions (out of 360, identified based on resting-state functional connectivity). The supplementary analysis was only performed on 153 regions, identified based on task-constrained co-activation maps, that were smaller in spatial extent than the resting-state functional connectivity. The differences between resting-state functional connectivity and task-constrained coactivations are illustrated in Figure S8: The coactivation maps, were less distinct across the thalamic seeds than the functional connectivity maps (see supplementary Figure S8). Particularly regions in sensorimotor cortex were more likely to be pre-selected based on task-constrained coactivations than based on resting state functional connectivity.

A comparison between both analyses revealed overall minor differences (see Supplementary Figures S9-11). The large autobiographical memory system, the nociception and the large finger tapping system were featured in both analyses. Notably, the autobiographical memory system featured more wide-spread thalamo-cortical associations in the main analysis, while the systems associated with somatosensory processes were more pronounced in the supplementary analysis. The more pronounced somatosensory results are a likely consequence of the more frequent pre-selection of somatosensory regions by task-constrained co-activations. Furthermore, the supplementary validation analysis revealed in total a larger number of different terms for each thalamic cluster. However, these terms were mostly anatomical or were associated with only a few thalamo-cortical pairs, which constraints their contribution to the general discussion of thalamo-cortical functioning (see supplementary Figures S10-12). In the light of these findings, the supplementary analysis gives us confidence that the systems-level decoding is useful to generate new hypotheses on seed-cortical functioning.

References:

Genon S, Li H, Fan L, et al (2016) The Right Dorsal Premotor Mosaic: Organization, Functions, and Connectivity. Cereb Cortex bhw065. https://doi.org/10.1093/cercor/bhw065

Supplementary Figure 8: The selected HCP-MMP regions and the knee of a curve values for each thalamic cluster of the systems-level decoding with the task-constraint coactivation maps. On the y-axis we plot the coverage (in percentage) of the HCP-cortical regions with the coactivation maps against all 360 cortical regions on the x-axis. Please note, the x-value of the knee of a curve represent the number of excluded cortical regions for each thalamic cluster. The left column shows the results for left clusters 1-4 (i.e., L1-L4), the right column the results for right clusters 1-3 (i.e., R1-R3) and at the right bottom we display all MACM-CBP derived thalamic clusters.

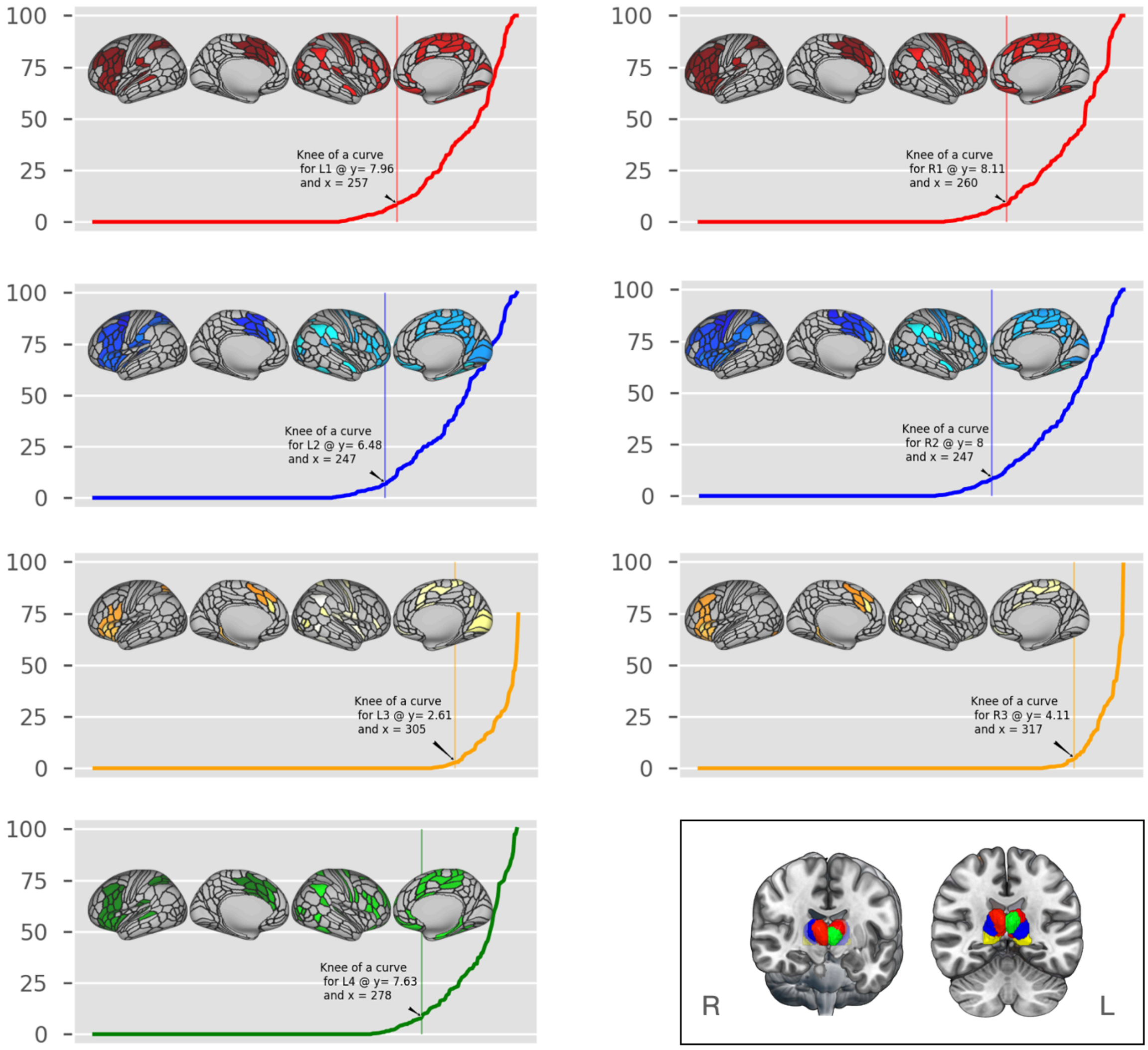

Supplementary Figure S9: Surface maps of the largest systems per thalamic cluster for the systems-level decoding of the task-constrained coactivation maps, overlaid with an outline grid representing the 360 HCP-MMP regions. The largest system is defined as the thalamo-cortical system with the highest percentage of co-activated cortical regions associated with a given term. Color gradings of the surface maps correspond to each thalamic cluster represented in the box on the bottom-left corner of the figure.

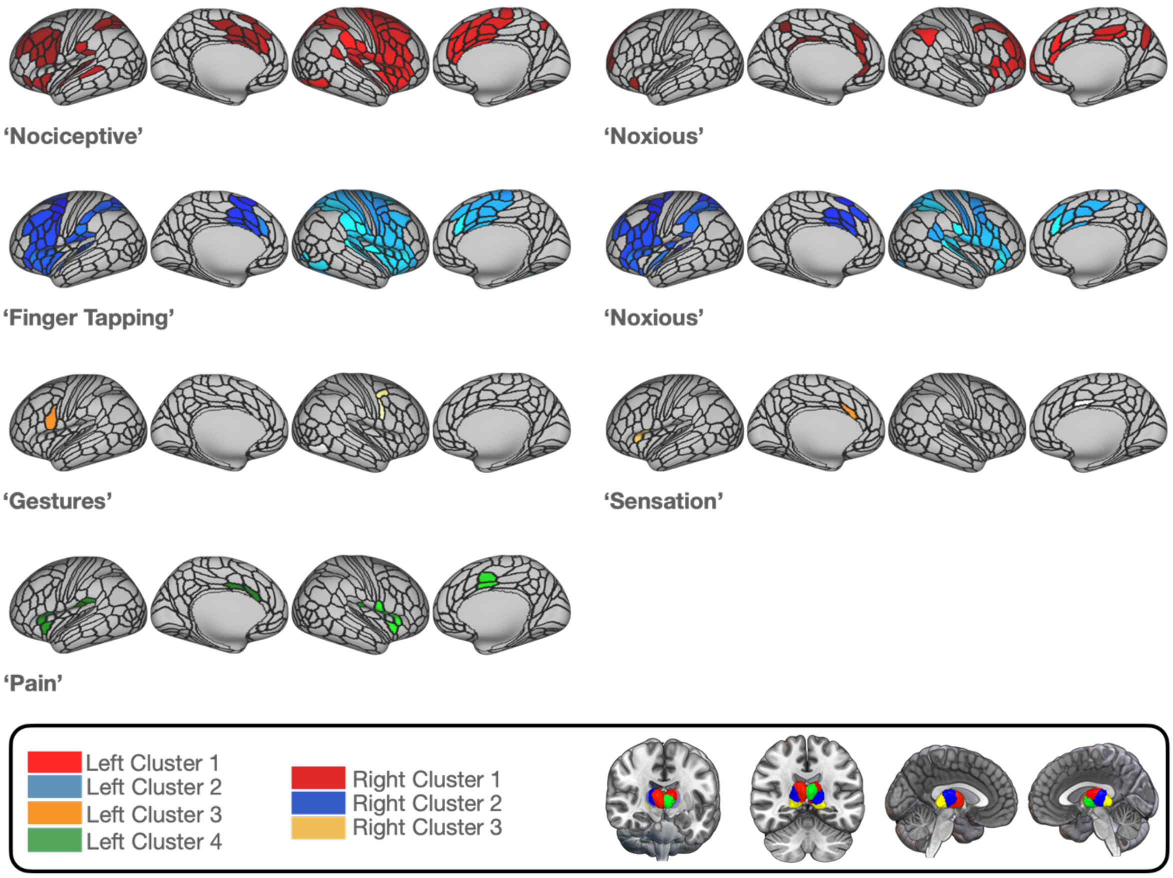

Supplementary Figure S10: Bar plots displaying the relative number of HCP-MMP cortical regions (i.e., num of cortical regions/360 *100) of the thalamo-cortical systems as they appear both, in the main analysis of the present study and as well in the supplementary validation of the systems-level decoding.

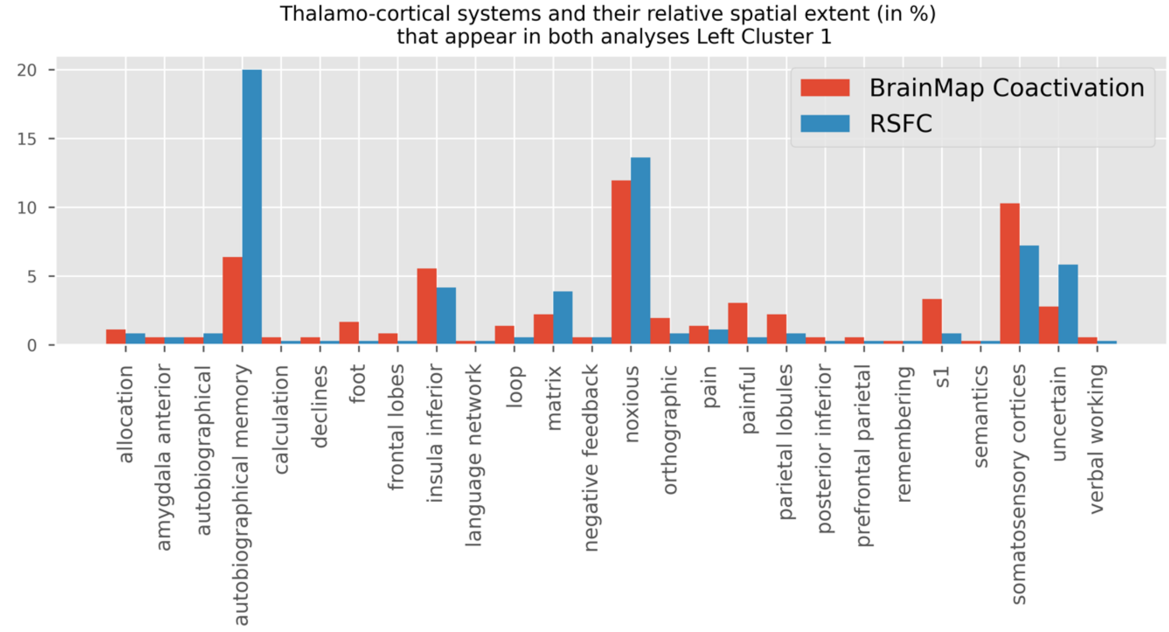
Thalamo-cortical systems and their relative spatial extent (in %)
that appear in both analyses Left Cluster 1
BrainMap Coactivation
RSFC
0
5
10
15
20
allocation
amygdala anterior
autobiographical
autobiographical memory
calculation
declines
foot
frontal lobes
insula inferior
language network
loop
matrix
negative feedback
noxious
orthographic
pain
painful
parietal lobules
posterior inferior
prefrontal parietal
remembering
s1
semantics
somatosensory cortices
uncertain
verbal working

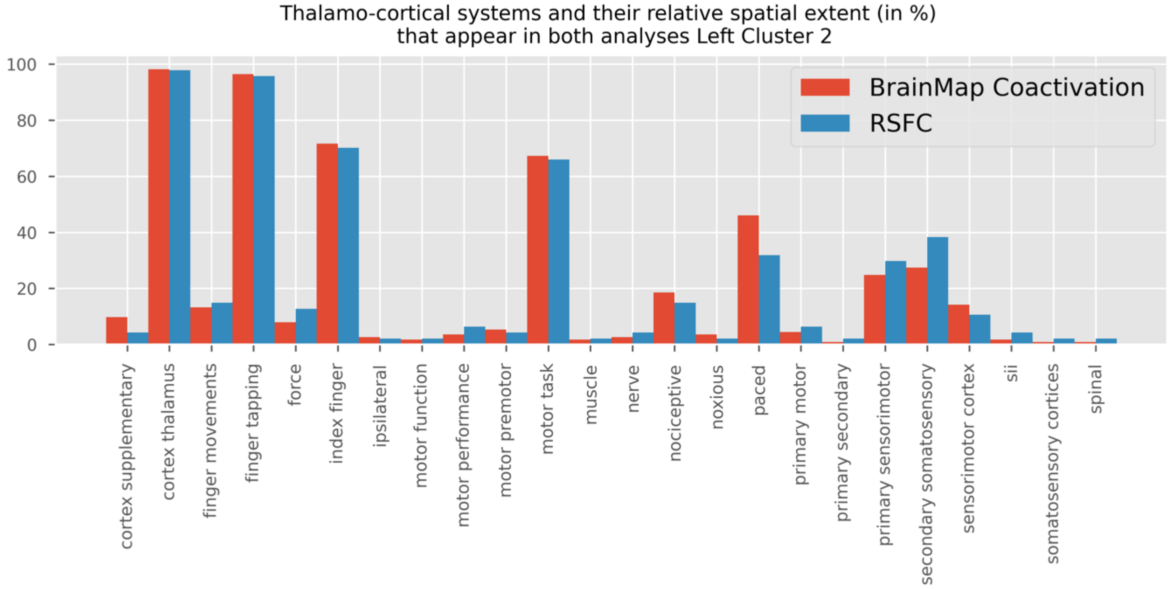
Thalamo-cortical systems and their relative spatial extent (in %)
that appear in both analyses Left Cluster 2
BrainMap Coactivation
RSFC
0
20
40
60
80
100
cortex supplementary
cortex thalamus
finger movements
finger tapping
force
index finger
ipsilateral
motor function
motor performance
motor premotor
motor task
muscle
nerve
nociceptive
noxious
paced
primary motor
primary secondary
primary sensorimotor
secondary somatosensory
sensorimotor cortex
sii
somatosensory cortices
spinal

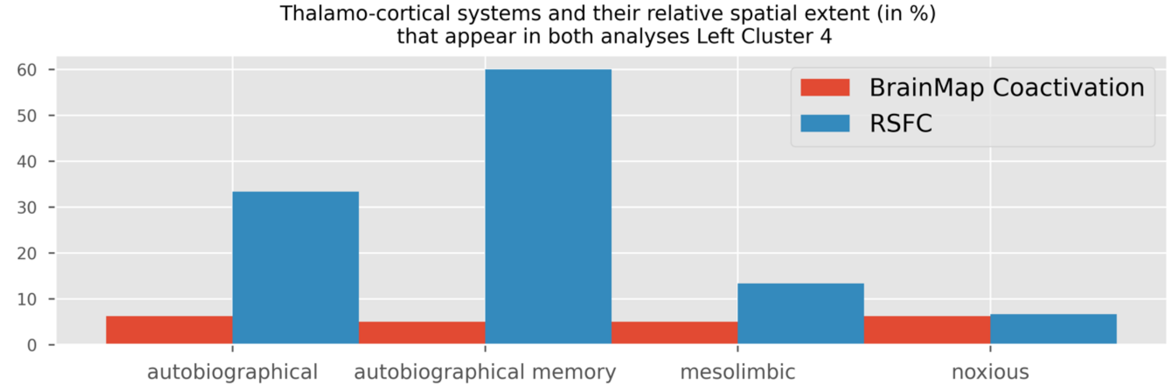
Thalamo-cortical systems and their relative spatial extent (in %)
that appear in both analyses Left Cluster 4
BrainMap Coactivation
RSFC
0
10
20
30
40
50
60
autobiographical
autobiographical memory
mesolimbic
noxious

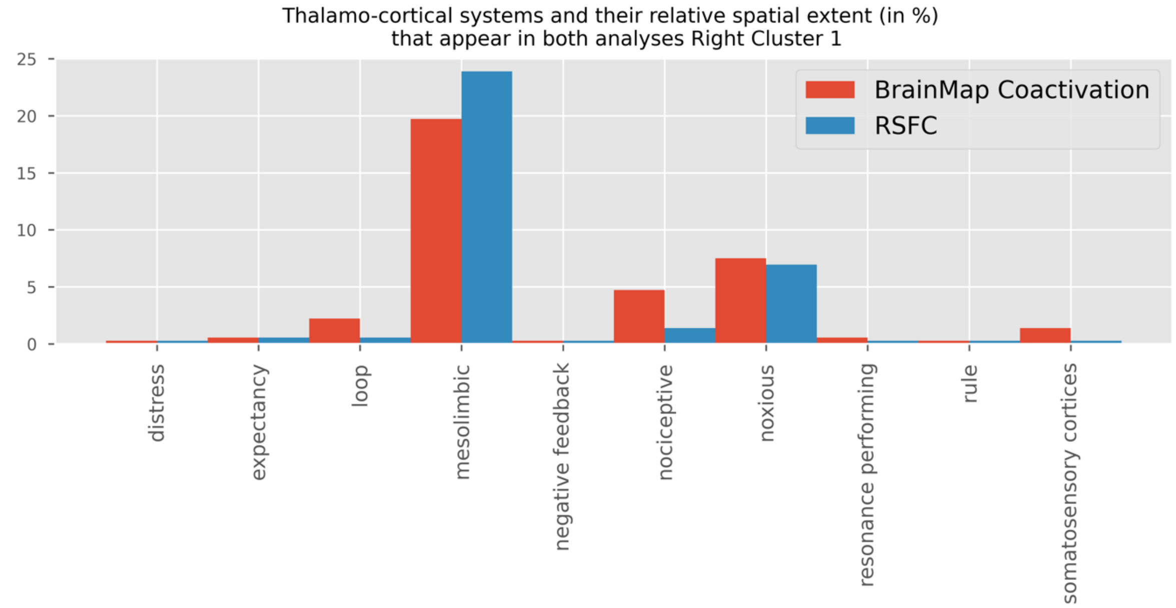

Thalamo-cortical systems and their relative spatial extent (in %)
that appear in both analyses Right Cluster 1
BrainMap Coactivation
RSFC
0
5
10
15
20
25
distress
expectancy
loop
mesolimbic
negative feedback
nociceptive
noxious
resonance performing
rule
somatosensory cortices

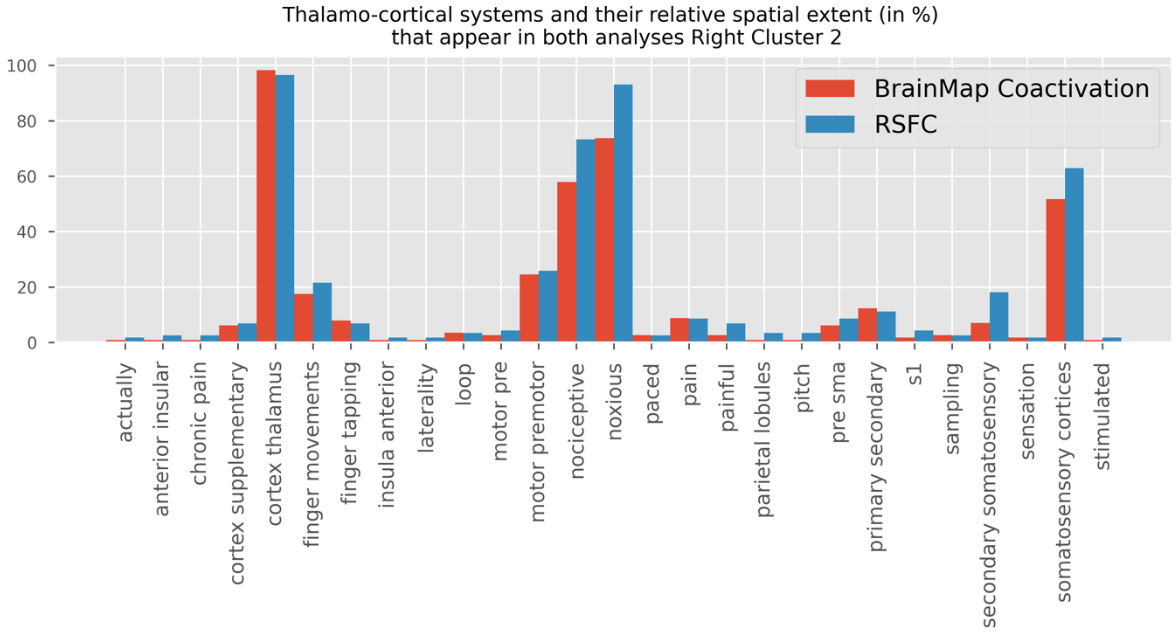

Thalamo-cortical systems and their relative spatial extent (in %)
that appear in both analyses Right Cluster 2
BrainMap Coactivation
RSFC
0
20
40
60
80
100
actually
anterior insular
chronic pain
cortex supplementary
cortex thalamus
finger movements
finger tapping
insula anterior
laterality
loop
motor pre
motor premotor
nociceptive
noxious
paced
pain
painful
parietal lobules
pitch
pre sma
primary secondary
s1
sampling
secondary somatosensory
sensation
somatosensory cortices
stimulated

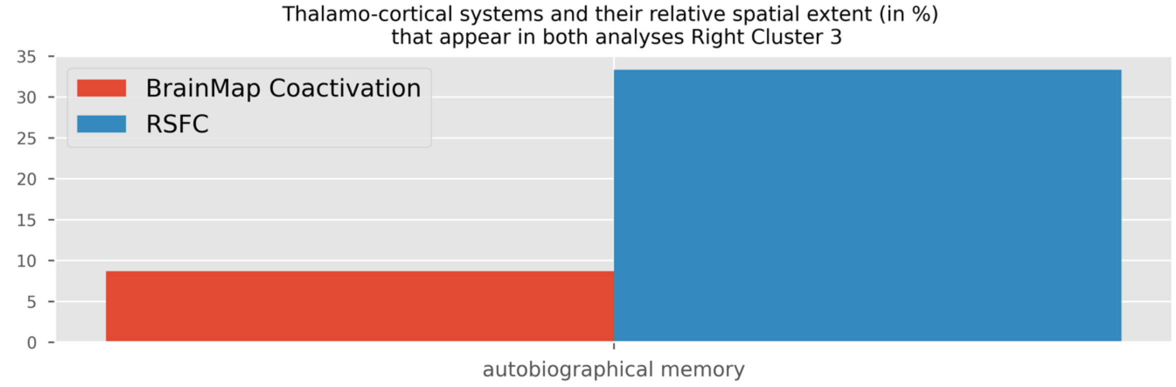

Thalamo-cortical systems and their relative spatial extent (in %)
that appear in both analyses Right Cluster 3
BrainMap Coactivation
RSFC
0
5
10
15
20
25
30
35
autobiographical memory

Supplementary Figure S11: Line plots of the thalamo-cortical systems and their spatial extent (in %) that appear only in the supplementary validation analysis of the systems-level decoding.

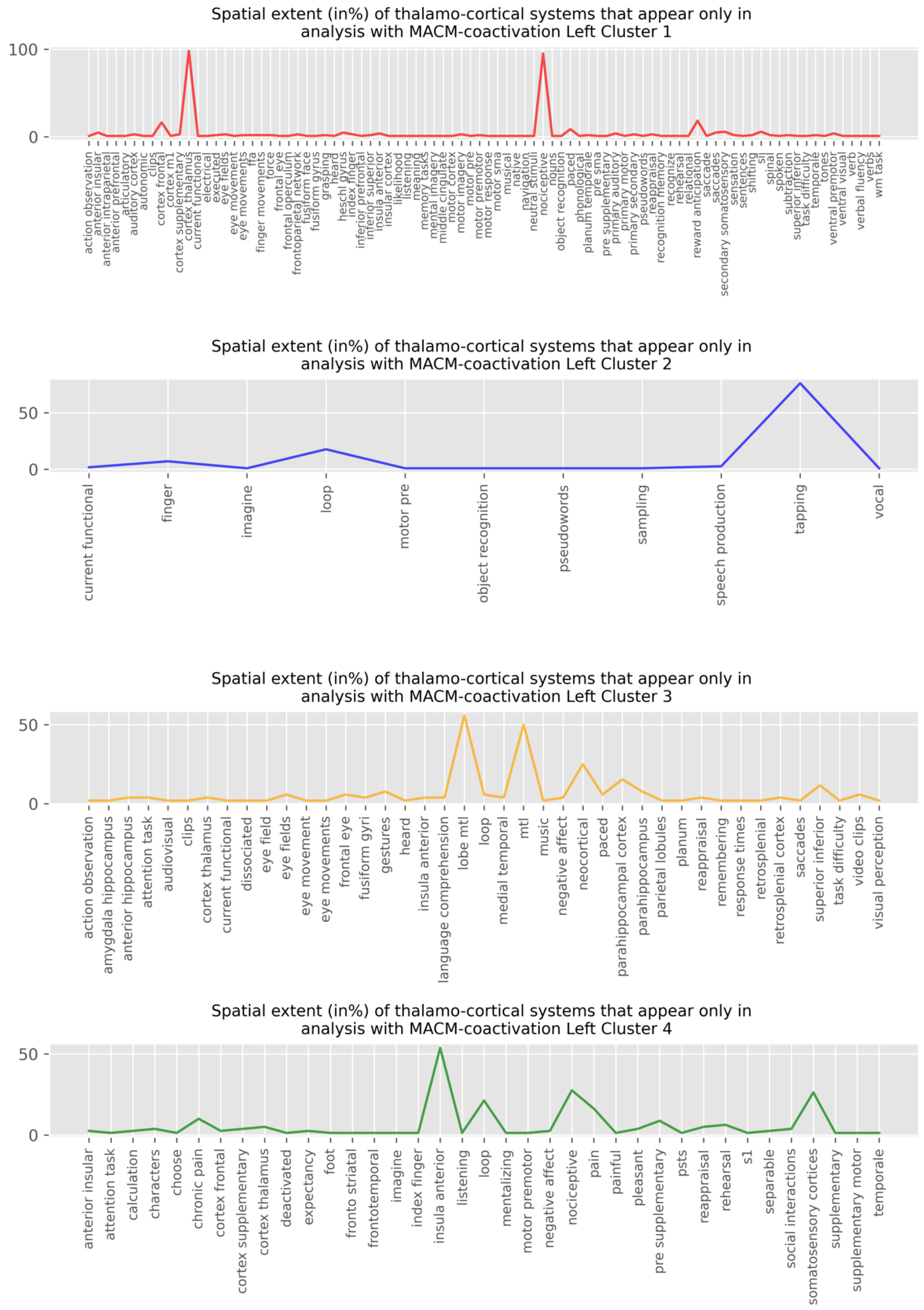

Spatial extent (in%) of thalamo-cortical systems that appear only in analysis with MACM-coactivation Right Cluster 1

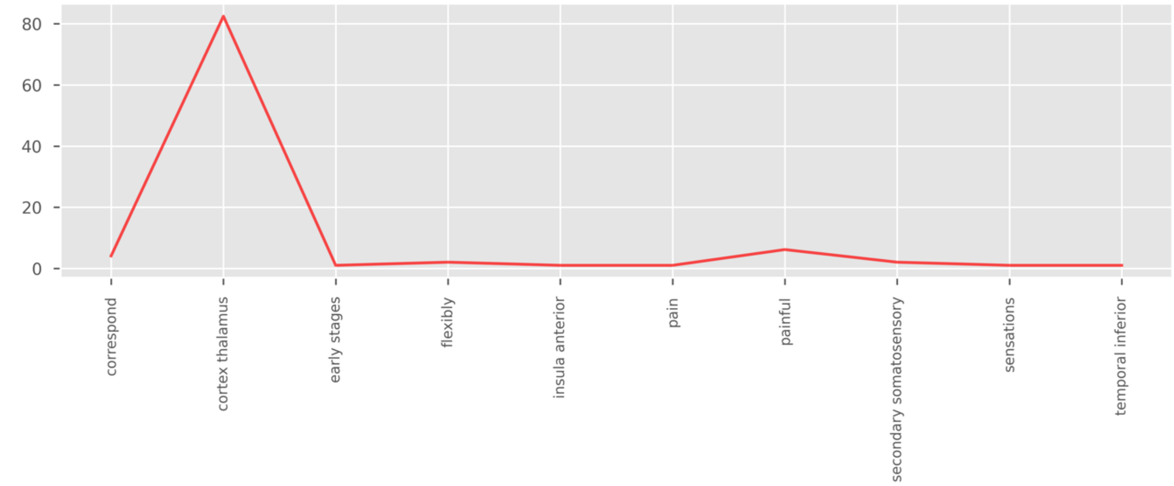

Spatial extent (in%) of thalamo-cortical systems that appear only in analysis with MACM-coactivation Right Cluster 2

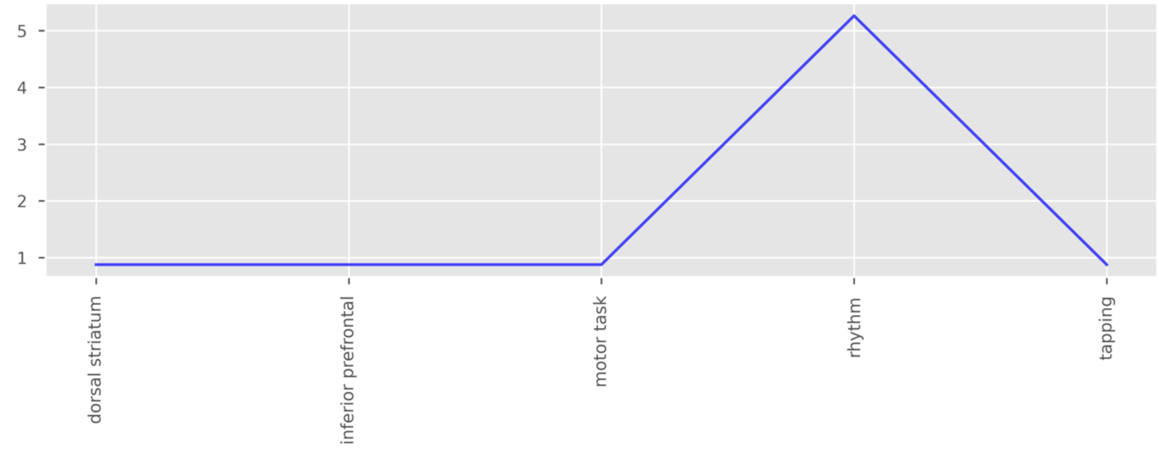

Spatial extent (in%) of thalamo-cortical systems that appear only in analysis with MACM-coactivation Right Cluster 3

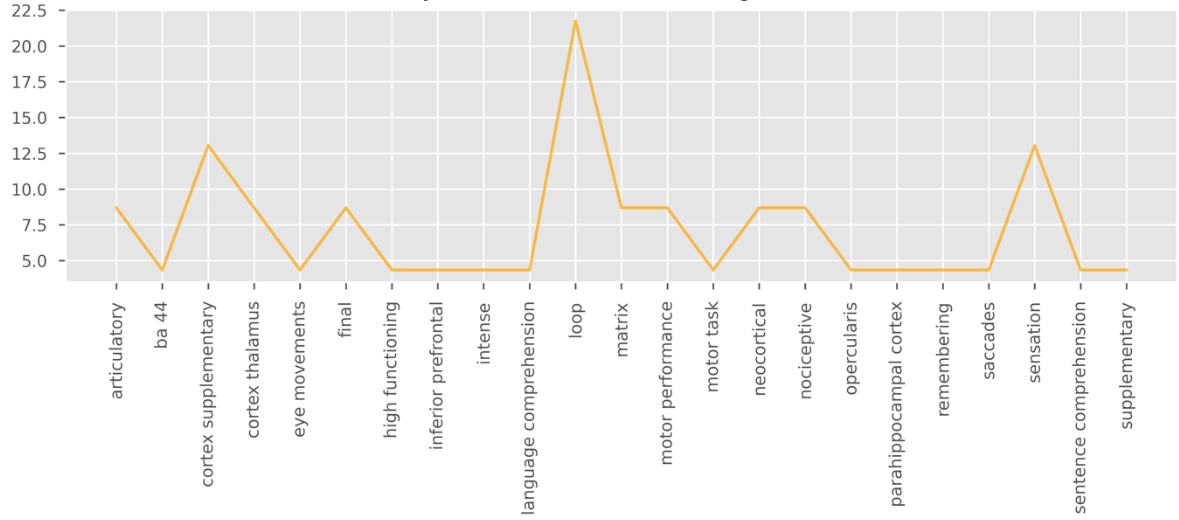

Supplementary Figure S12: Line plots of the thalamo-cortical systems and their spatial extent (in %) that appear only in the main analysis of the systems-level decoding. Please note, left cluster 2 featured no unique terms, left cluster 3 and left cluster 4 featured only a single unique term (i.e., ‘memory test’ and ‘periaqueductal”) and right cluster 3 two unique terms (‘autobiographical’, ‘scenes’).

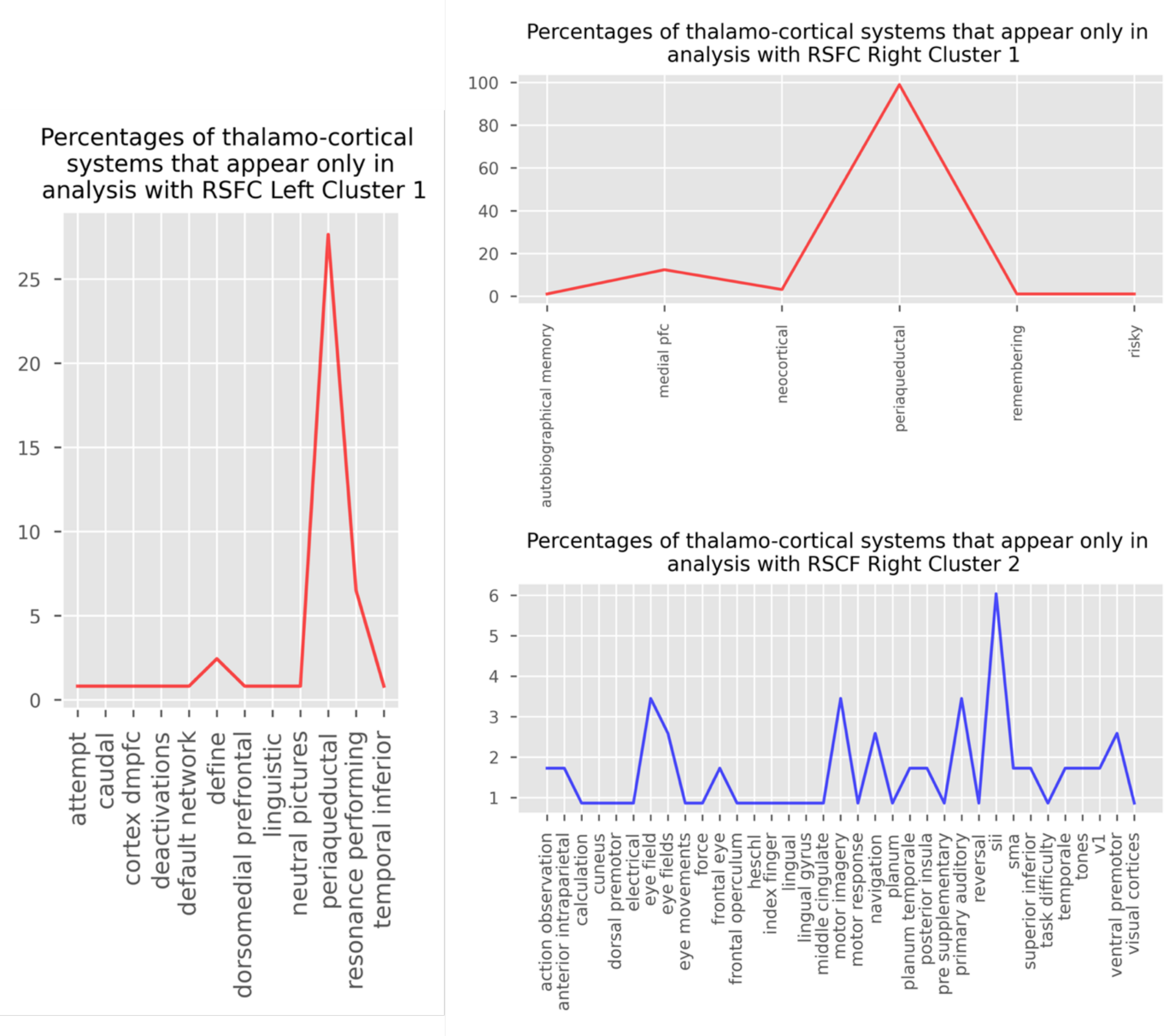

Supplementary Table S13: Jaccard index overlaps of the MACM-CBP derived thalamic clusters with the Morel atlas template.

Thalamic Group	Thalamic Nuclei	Abbr.	MACM-CBP derived Thalamic Clusters						
			Left Cluster 1	Left Cluster 2	Left Cluster 3	Left Cluster 4	Right Cluster 1	Right Cluster 2	Right Cluster 3
Medial Group									
	Mediodorsal nucleus	MD	0	0	0	0	0	0	0
	magnocellular	MDmc	0	0	0	0	0	0	0
	parvocellular	MDpc	0.15	0	0	0.13	0.2	0	0
	Medioventral nucleus	MV	0	0	0	0	0	0	0
	Central lateral nucleus	CL	0.15	0	0	0	0.1	0.1	0
	Central medial Nucleus	CeM	0	0	0	0	0	0	0
	Centromedian nucleus	CM	0	0.11	0	0	0	0	0
	Paraventricular nucleus	Pv	0	0	0	0	0	0	0
	Habenular nucleus	Hb	0	0	0	0	0	0	0
	Parafascicular nucleus	Pf	0	0	0	0	0	0	0
	Subparafascicular nulceus	sPf	0	0	0	0	0	0	0
Posterior group									
	Medial pulvinar	PuM	0	0.13	0.44	0	0	0	0.56
	Inferior pulvinar	PuI	0	0	0	0	0	0	0
	Lateral pulvinar	PuL	0	0	0	0	0	0	0
	Anterior pulvinar	PuA	0	0	0	0	0	0	0
	Lateral posterior nucleus	LP	0	0.12	0	0	0	0.16	0
	Medial geniculate nucleus	MGN	0	0	0	0	0	0	0
	Suprageniculate nucleus	SG	0	0	0	0	0	0	0
	Limitans nucleus	Li	0	0	0	0	0	0	0
	Posterior nucleus	Po	0	0	0	0	0	0	0
	Lateral geniculate nucleus	LGN	0	0	0	0	0	0	0
Lateral group									
	Ventral posterior lateral nucleus	VPL	0	0	0	0	0	0	0

	Anterior part	VPLa	0	0	0	0	0	0	0
	Posterior part	VPLp	0	0.15	0	0	0	0.14	0
	Ventral posterior medial nucleus	VPM	0	0	0	0	0	0	0
	Ventral posterior inferior nucleus	VPI	0	0	0	0	0	0	0
	Ventral lateral nucleus	VL	0	0	0	0	0	0	0
	Ventral lateral anterior nucleus	VLa	0	0	0	0	0	0	0
	Ventral lateral posterior nucleus	VLp	0	0	0	0	0	0	0
	Dorsal part	VLpd	0.2	0	0	0	0	0.13	0
	Ventral part	VLpv	0	0	0	0	0	0.12	0
	Ventral anterior nucleus	VA	0	0	0	0	0	0	0
	Magnocellular part	VAmc	0	0	0	0	0	0	0
	Parvocellular part	VApc	0	0	0	0.14	0.13	0	0
	Ventral medial nucleus	VM	0	0	0	0	0	0	0
Anterior group									
	Anterior dorsal nucleus	AD	0	0	0	0	0	0	0
	Anterior medial nucleus	AM	0	0	0	0	0	0	0
	Anterior ventral nucleus	AV	0	0	0	0	0	0	0
	Lateral dorsal nucleus	LD	0	0	0	0	0	0	0
Other structures									
	Red nucleus	RN	0	0	0	0	0	0	0
	Mammillothalamic tract	mtt	0	0	0	0	0	0	0
	Subthalamic nucleus	STh	0	0	0	0	0	0	0

Supplementary Figure S14: Axial slices of the Morel thalamic nuclei divided over the major thalamic nuclei groups.

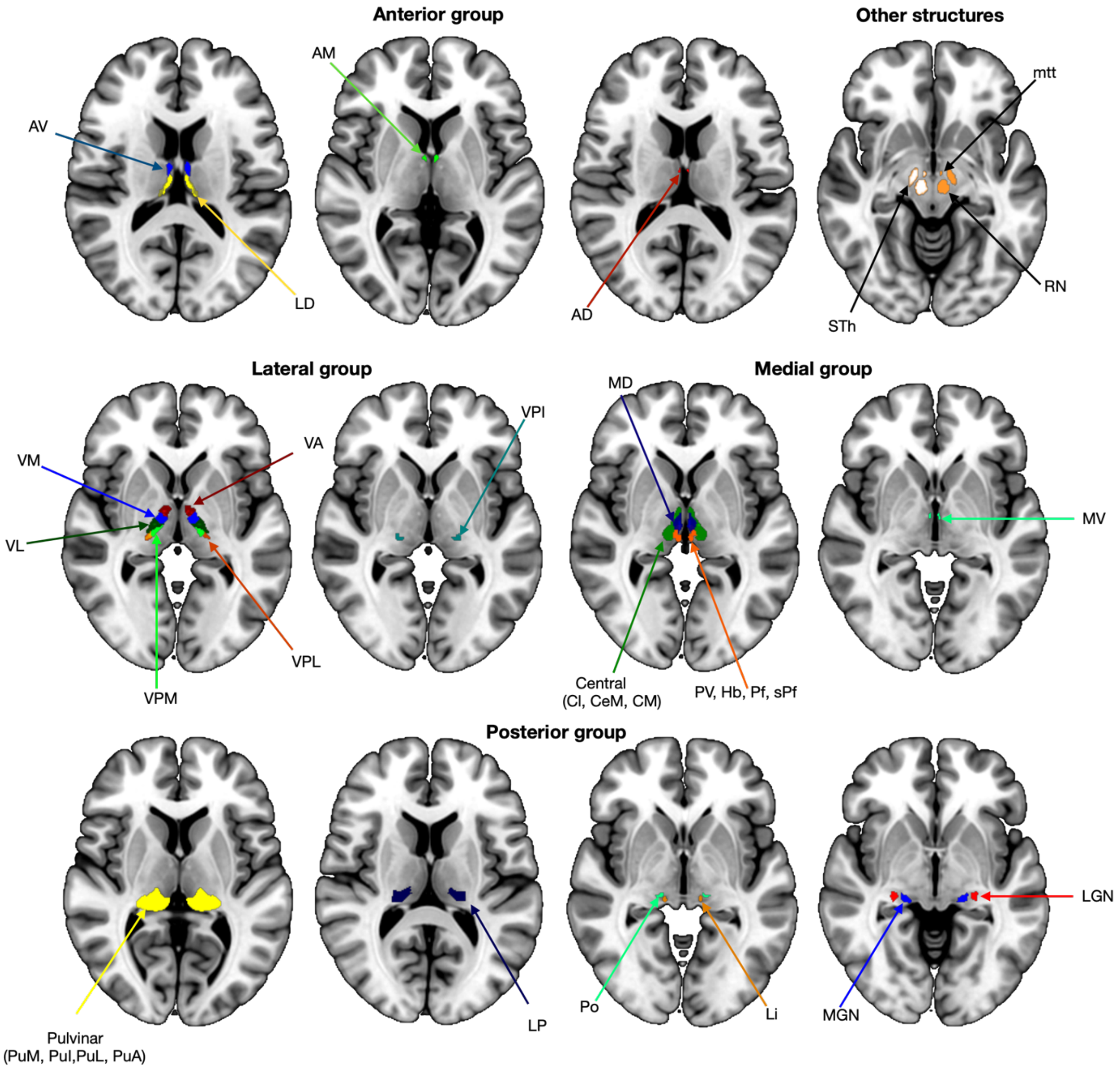

Supplementary Table S15: Top 10 terms with the highest posterior probability (i.e., probReverse) of the standard Bayesian reverse inference decoding of the thalamic clusters.

Thalamic clusters	Terms	pReverse	zReverse	probReverse
**Left Cluster 1**	cortex thalamus	1.38E-11	6.76	0.79
	thalamus	3.93E-73	18.09	0.77
	nociceptive	1.12E-06	4.87	0.77
	thalamic	2.51E-16	8.19	0.75
	incentive delay	0.002	3.06	0.73
	periaqueductal	0.003	2.99	0.72
	autobiographical memory	0.01	2.74	0.71
	reward anticipation	0.01	2.70	0.71
	cortex frontal	0.02	2.25	0.71
	somatosensory cortices	0.01	2.74	0.70
**Left cluster 2**	cortex thalamus	1.23E-20	9.31	0.83
	finger tapping	3.90E-10	6.26	0.79
	thalamus	3.32E-86	19.68	0.79
	index finger	1.20E-06	4.86	0.77
	tapping	1.99E-10	6.36	0.76
	motor task	4.77E-09	5.85	0.76
	thalamic	5.46E-16	8.10	0.75
	primary sensorimotor	2.55E-06	4.70	0.75
	paced	3.76E-06	4.62	0.74
	secondary somatosensory	4.62E-09	5.86	0.74
**Left cluster 3**	lobe mtl	4.91E-08	5.45	0.81
	mtl	2.22E-09	5.98	0.81
	neocortical	0.01	2.66	0.78
	word pairs	0.01	2.66	0.78
	parahippocampal cortex	0.01	2.53	0.77
	designs	0.01	2.49	0.77
	direct evidence	0.01	2.69	0.77
	confidence	0.02	2.40	0.77
	mesial	0.01	2.48	0.76

	retrosplenial cortex	0.04	2.03	0.75
**Left Cluster 4**	thalamus	1.36E-60	16.42	0.80
	sexual	0.0001	3.80	0.78
	vivo	0.0002	3.77	0.77
	hypothalamus	0.0001	3.87	0.77
	incentive delay	0.01	2.78	0.75
	ventral tegmental	0.01	2.44	0.74
	tegmental	0.02	2.40	0.74
	somatosensory cortices	0.003	2.97	0.74
	chronic pain	0.01	2.64	0.74
	loop	0.02	2.27	0.74
**Right cluster 1**	thalamic	3.93E-30	11.41	0.78
	thalamus	5.11E-81	19.06	0.78
	periaqueductal	9.69E-09	5.74	0.77
	hypothalamus	2.07E-08	5.61	0.76
	mesolimbic	3.47E-05	4.14	0.74
	cortex thalamus	1.45E-05	4.34	0.74
	incentive delay	0.0002	3.70	0.73
	nucleus accumbens	2E-10	6.39	0.73
	midbrain	1.35E-10	6.42	0.73
	monetary reward	3.47E-05	4.14	0.73
**Right cluster 2**	cortex thalamus	4.63E-21	9.42	0.82
	thalamus	2.47E-97	20.94	0.79
	thalamic	6.38E-26	10.53	0.78
	noxious	8.28E-10	6.14	0.78
	nociceptive	9.02E-05	3.92	0.74
	periaqueductal	0.0001	3.85	0.74
	somatosensory cortices	4.61E-06	4.58	0.74
	nuclei	5.99E-08	5.42	0.73
	ganglia	2.22E-22	9.73	0.73
	basal ganglia	5.58E-22	9.64	0.73
**Right**	group healthy	0.01	2.65	0.78

**cluster 3**	neocortical	0.02	2.36	0.78
	phenomena	0.05	1.99	0.76
	sampling	0.09	1.71	0.76
	coordination	0.00	3.16	0.76
	final	0.06	1.88	0.75
	thalamus	5.49E-21	9.40	0.75
	parahippocampal cortex	0.11	1.61	0.75
	advanced	0.09	1.71	0.75
	cortex thalamus	0.09	1.69	0.75

Supplementary Table S16: Systems Decoding terms, percentages, number of joint cortical regions, the mean, standard deviation, max and min value of the Bayes Factors associated with the term for each thalamic cluster. The Bayes Factors were calculated based on the ratio between the posterior odds (reverse Probability, thus probReverse of a term) and the prior odds (prior odds = 0.5) (ref. Goodman 1999; Poldrack 2006).

Thalamic Clusters	Terms	Percentages	Number of regions	Bayes Factor mean	Stdev	Max	Min
**Left cluster 1**	allocation	2.44	3	2.267	0.164	2.42	2.094
	amygdala anterior	1.63	2	2.118	0.036	2.143	2.092
	attempt	0.81	1	2.102		2.102	2.102
	autobiographical	2.44	3	2.053	0.1	2.163	1.968
	autobiographical memory	58.54	72	2.386	0.18	2.788	1.915
	calculation	0.81	1	2.007		2.007	2.007
	caudal	0.81	1	2.098		2.098	2.098
	cortex dmpfc	0.81	1	2.052		2.052	2.052
	deactivations	0.81	1	2.038		2.038	2.038
	declines	0.81	1	2.404		2.404	2.404
	default network	0.81	1	2.333		2.333	2.333
	define	2.44	3	2.185	0.116	2.281	2.055
	dorsomedial prefrontal	0.81	1	2.058		2.058	2.058
	foot	0.81	1	2.078		2.078	2.078

	frontal lobes	0.81	1	2.055		2.055	2.055
	insula inferior	12.20	15	2.263	0.067	2.382	2.125
	language network	0.81	1	2.111		2.111	2.111
	linguistic	0.81	1	1.865		1.865	1.865
	loop	1.63	2	2.023	0.075	2.076	1.969
	matrix	11.38	14	2.32	0.121	2.512	2.144
	negative feedback	1.63	2	2.096	0.102	2.168	2.024
	neutral pictures	0.81	1	2.079		2.079	2.079
	noxious	39.84	49	2.282	0.129	2.656	1.981
	orthographic	2.44	3	2.105	0.079	2.195	2.046
	pain	3.25	4	2.214	0.143	2.367	2.01
	painful	1.63	2	2.18	0.135	2.276	2.084
	parietal lobules	2.44	3	2.153	0.217	2.372	1.938
	periaqueductal	27.64	34	2.327	0.121	2.813	2.125
	posterior inferior	0.81	1	2.101		2.101	2.101
	prefrontal parietal	0.81	1	2.104		2.104	2.104
	remembering	0.81	1	1.984		1.984	1.984
	resonance performing	6.50	8	2.116	0.06	2.213	2.009
	s1	2.44	3	2.361	0.298	2.659	2.064
	semantics	0.81	1	2.008		2.008	2.008
	somatosensory cortices	21.14	26	2.205	0.151	2.487	1.924
	temporal inferior	0.81	1	2.111		2.111	2.111
	uncertain	17.07	21	2.267	0.167	2.622	1.872
	verbal working	0.81	1	2.153		2.153	2.153
**Left cluster 2**	cortex supplementary	4.26	2	2.356	0.103	2.429	2.283
	cortex thalamus	97.87	46	3.909	0.476	4.769	2.562
	finger movements	14.89	7	2.688	0.242	2.987	2.277
	finger tapping	95.74	45	3.184	0.371	3.709	2.464
	force	12.77	6	2.798	0.231	3.025	2.458
	index finger	70.21	33	3.012	0.27	3.443	2.416
	ipsilateral	2.13	1	2.642		2.642	2.642
	motor function	2.13	1	2.473		2.473	2.473
	motor performance	6.38	3	2.821	0.063	2.892	2.771

	motor premotor	4.26	2	2.909	0.097	2.978	2.84
	motor task	65.96	31	2.816	0.233	3.125	2.273
	muscle	2.13	1	2.499		2.499	2.499
	nerve	4.26	2	2.974	0.207	3.121	2.828
	nociceptive	14.89	7	2.747	0.177	3.09	2.587
	noxious	2.13	1	2.897		2.897	2.897
	paced	31.91	15	2.732	0.247	3.025	2.287
	primary motor	6.38	3	2.668	0.286	2.874	2.342
	primary secondary	2.13	1	2.578		2.578	2.578
	primary sensorimotor	29.79	14	2.792	0.216	3.069	2.314
	secondary somatosensory	38.30	18	2.772	0.102	2.9	2.667
	sensorimotor cortex	10.64	5	2.588	0.116	2.67	2.506
	sii	4.26	2	2.782		2.782	2.782
	somatosensory cortices	2.13	1	2.773		2.773	2.773
	spinal	2.13	1	2.356		2.429	2.283
**Left cluster 3**	memory test	100.00	2				
**Left cluster 4**	autobiographical	33.33	5	2.421	0.115	2.564	2.265
	autobiographical memory	60.00	9	2.45	0.117	2.683	2.273
	mesolimbic	13.33	2	2.505	0.034	2.529	2.481
	noxious	6.67	1	2.389		2.389	2.389
	periaqueductal	80.00	12	2.665	0.167	3.016	2.484
**Right cluster 1**	autobiographical memory	1.03	1	2.237		2.237	2.237
	distress	1.03	1	1.921		1.921	1.921
	expectancy	2.06	2	1.991	0.109	2.068	1.914
	loop	2.06	2	2.094	0.029	2.115	2.074
	medial pfc	12.37	12	2.257	0.193	2.431	1.854
	mesolimbic	88.66	86	2.535	0.207	2.964	2.007
	negative feedback	1.03	1	2.227		2.227	2.227
	neocortical	3.09	3	2.281	0.11	2.394	2.175
	nociceptive	5.15	5	2.257	0.238	2.582	1.974
	noxious	25.77	25	2.392	0.194	2.72	1.931
	periaqueductal	98.97	96	2.771	0.3	3.573	1.959
	remembering	1.03	1	2.011		2.011	2.011

	resonance performing	1.03	1	2.135		2.135	2.135
	risky	1.03	1	2.208		2.208	2.208
	rule	1.03	1	1.89		1.89	1.89
	somatosensory cortices	1.03	1	2.346		2.346	2.346
**Right cluster 2**	action observation	1.72	2	2.36	0.069	2.409	2.311
	actually	1.72	2	2.302	0.015	2.313	2.291
	anterior insular	2.59	3	2.472	0.244	2.666	2.198
	anterior intraparietal	1.72	2	2.53	0.235	2.696	2.364
	calculation	0.86	1	2.121		2.121	2.121
	chronic pain	2.59	3	2.543	0.186	2.747	2.383
	cortex supplementary	6.90	8	2.451	0.124	2.579	2.246
	cortex thalamus	96.55	112	3.835	0.453	4.608	2.568
	cuneus	0.86	1	1.947		1.947	1.947
	dorsal premotor	0.86	1	2.54		2.54	2.54
	electrical	0.86	1	2.534		2.534	2.534
	eye field	3.45	4	2.82	0.456	3.334	2.281
	eye fields	2.59	3	2.548	0.237	2.715	2.38
	eye movements	0.86	1	2.339		2.339	2.339
	finger movements	21.55	25	2.537	0.239	3.257	2.121
	finger tapping	6.90	8	2.382	0.135	2.532	2.129
	force	0.86	1	2.253		2.253	2.253
	frontal eye	1.72	2	2.733	0.103	2.806	2.66
	frontal operculum	0.86	1	2.602		2.602	2.602
	heschl	0.86	1	2.396		2.396	2.396
	index finger	0.86	1	2.562		2.562	2.562
	insula anterior	1.72	2	2.461	0.291	2.667	2.255
	laterality	1.72	2	2.247	0.175	2.371	2.123
	lingual	0.86	1	1.87		1.87	1.87
	lingual gyrus	0.86	1	1.877		1.877	1.877
	loop	3.45	4	2.241	0.097	2.354	2.125
	middle cingulate	0.86	1	2.598		2.598	2.598
	motor imagery	3.45	4	2.285	0.161	2.494	2.151
	motor pre	4.31	5	2.333	0.113	2.449	2.184

	motor premotor	25.86	30	2.469	0.243	2.92	1.942
	motor response	0.86	1	2.306		2.306	2.306
	navigation	2.59	3	2.026	0.057	2.091	1.986
	nociceptive	73.28	85	2.637	0.285	3.516	2.053
	noxious	93.10	108	2.92	0.359	3.609	2.033
	paced	2.59	3	2.368	0.371	2.713	1.976
	pain	8.62	10	2.525	0.169	2.859	2.154
	painful	6.90	8	2.548	0.17	2.844	2.3
	parietal lobules	3.45	4	2.242	0.066	2.31	2.153
	pitch	3.45	4	2.513	0.183	2.78	2.367
	planum	0.86	1	2.82		2.82	2.82
	planum temporale	1.72	2	2.682	0.259	2.865	2.499
	posterior insula	1.72	2	2.622	0.212	2.772	2.472
	pre sma	8.62	10	2.25	0.205	2.5	1.851
	pre supplementary	0.86	1	2.071		2.071	2.071
	primary auditory	3.45	4	2.571	0.105	2.667	2.471
	primary secondary	11.21	13	2.561	0.192	2.901	2.282
	reversal	0.86	1	2.174		2.174	2.174
	s1	4.31	5	2.73	0.303	3.205	2.449
	sampling	2.59	3	2.514	0.113	2.592	2.384
	secondary somatosensory	18.10	21	2.628	0.245	3.16	2.141
	sensation	1.72	2	2.39	0.128	2.481	2.299
	sii	6.03	7	2.753	0.226	3.113	2.54
	sma	1.72	2	2.24	0.093	2.306	2.175
	somatosensory cortices	62.93	73	2.609	0.294	3.618	2.005
	stimulated	1.72	2	2.431	0.034	2.455	2.407
	superior inferior	1.72	2	2.035	0.123	2.122	1.948
	task difficulty	0.86	1	2.055		2.055	2.055
	temporale	1.72	2	2.682	0.211	2.865	2.499
	tones	1.72	2	2.515	0.136	2.61	2.419
	v1	1.72	2	2.219	0.227	2.38	2.059
	ventral premotor	2.59	3	2.469	0.119	2.596	2.359
	visual cortices	0.86	1	2.369		2.369	2.369

**Right cluster 3**	autobiographical	66.67	2	2.735	0.182	2.863	2.606
	autobiographical memory	33.33	1	3.026		3.026	3.026
	scenes	33.33	1	2.357		2.357	2.357

References:

Goodman SN (1999) Toward Evidence-Based Medical Statistics. 2: The Bayes Factor. Ann Intern Med 130:1005. https://doi.org/10.7326/0003-4819-130-12-199906150-00019

Poldrack R (2006) Can cognitive processes be inferred from neuroimaging data? Trends Cogn Sci 10:59-63 https://doi.org/10.1016/j.tics.2005.12.004

Supplementary Table S17: All terms of the supplementary analysis per thalamic cluster.

**Left cluster 1**	**Left cluster 2**	**Left cluster 3**	**Left cluster 4**	**Right cluster 1**	**Right cluster 2**	**Right cluster 3**
chosen	globus	anterior hippocampus	chronic pain	accumbens	basal	advanced
chronic pain	globus pallidus	confidence	cortex thalamus	anterior medial	basal ganglia	anterior medial
cortex frontal	motor network	direct evidence	daily life	correspond	coordination	certain
cortex thalamus	oral	episodic	hypothalamu s	cortex thalamus	cortical subcortical	clips
cortical subcortical	pallidus	episodic memory	incentive delay	cortical subcortical	dorsal striatum	cognitive task
deprivation	sampling	lobe mtl	insula anterior	cortico	early stage	controversial
drugs	secondary	mtl	loop	early stages	ganglia	coordination
eighteen	tapping	neocortical	men women	hypoactivation	inducing	depicting
equally	thalamic	occipital temporal	midbrain	hypothalamus	integrate	differentiate
hypoactivatio n	thalamus	parahippocamp al cortex	nociceptive	incentive delay	nuclei	final
impacts	ventral tegmental	retrosplenial cortex	personality traits	losses	periaqueductal	functional connections
incentive		single subject	reappraisal	midbrain	requirements	group healthy
incentive delay		thalamic	reward anticipation	monetary incentive	rhythm	middle occipital
indirect		video clips	semantic memory	monetary reward	tapping	mild cognitive

lobules		word pairs	sexual	nucleus accumbens	tesla	neocortical
monetary incentive			social interaction	secondary somatosensory	thalamic	parahippocampal cortex
networks involved			somatosensory cortices	seeking	thalamus	phenomena
nociceptive			tegmental	sexual	time task	precuneus posterior
nuclei			thalamic	social interaction	vermis	sampling
paced			thalamus	substantia		spectrum disorder
preparation			trigger	tegmental		temporoparietal
reward anticipation			unpleasant	thalamic		thalamus
share			ventral tegmental	thalamus		
storage				ventral tegmental		
structures involved						
task conditions						
task task						
thalamic						
thalamus						
transition						
